# S1PR1/S1PR3-YAP signaling and S1P-ALOX15 signaling contribute to an aggressive behavior in obesity-lymphoma

**DOI:** 10.1186/s13046-022-02589-7

**Published:** 2023-01-05

**Authors:** Xingtong Wang, Wei Guo, Xiaoju Shi, Yujia Chen, Youxi Yu, Beibei Du, Min Tan, Li Tong, Anna Wang, Xianying Yin, Jing Guo, Robert C. Martin, Ou Bai, Yan Li

**Affiliations:** 1 Department of Surgery, School of Medicine, University of Louisville, 511 S Floyd ST MDR Bldg Rm326A, Louisville, KY 40202 USA; 2 Department of Hematology, Cancer Center, The First Hospital of Jilin University, No. 71. Xinmin Street, Changchun, 130021 Jilin China; 3 Department of Hepatobiliary and Pancreatic Surgery, The First Hospital of Jilin University, Changchun, 130021 China; 4 Department of Gastrointestinal Surgery, The First Hospital of Jilin University, Changchun, 130021 China; 5 Department of Cardiology, China-Japan Union hospital of Jilin University, Changchun, 130033 China; 6 Department of Epidemiology and Biostatistics, School of Public Health, Jilin University, Changchun, 130021 China

**Keywords:** Lymphoma, Free fatty acid, S1P/SPHK signaling, Tumor microenvironment, Obesity

## Abstract

**Background:**

Excess body weight has been found to associate with an increased risk of lymphomas and some metabolic pathways are currently recognized in lymphomagenesis. Bioactive lipid metabolites such as sphingosine-1-phosphate (S1P) have been proposed to play an important role linking obesity and lymphomas. However, the underlying mechanism(s) of S1P signaling in obesity-lymphomagenesis have not been well addressed.

**Methods:**

The gene expression of sphingosine kinase (SPHK), lymphoma prognosis, and S1P production were analyzed using Gene Expression Omnibus (GEO) and human lymphoma tissue array. Obesity-lymphoma mouse models and lymphoma cell lines were used to investigate the S1P/SPHK-YAP axis contributing to obesity-lymphomagenesis. By using the mouse models and a monocyte cell line, S1P-mediated polarization of macrophages in the tumor microenvironment were investigated.

**Results:**

In human study, up-regulated S1P/SPHK1 was found in human lymphomas, while obesity negatively impacted progression-free survival and overall survival in lymphoma patients. In animal study, obesity-lymphoma mice showed an aggressive tumor growth pattern. Both in vivo and in vitro data suggested the existence of S1P-YAP axis in lymphoma cells, while the S1P-ALOX15 signaling mediated macrophage polarization towards TAMs exacerbated the lymphomagenesis. In addition, treatment with resveratrol in obesity-lymphoma mice showed profound effects of anti-lymphomagenesis, via down-regulating S1P-YAP axis and modulating polarization of macrophages.

**Conclusion:**

S1P/S1PR initiated the feedback loops, whereby S1P-S1PR1/S1PR3-YAP signaling mediated lymphomagenesis contributing to tumor aggressive growth, while S1P-ALOX15 signaling mediated TAMs contributing to immunosuppressive microenvironment in obesity-lymphoma. S1P-targeted therapy could be potentially effective and immune-enhancive against obesity-lymphomagenesis.

**Graphical Abstract:**

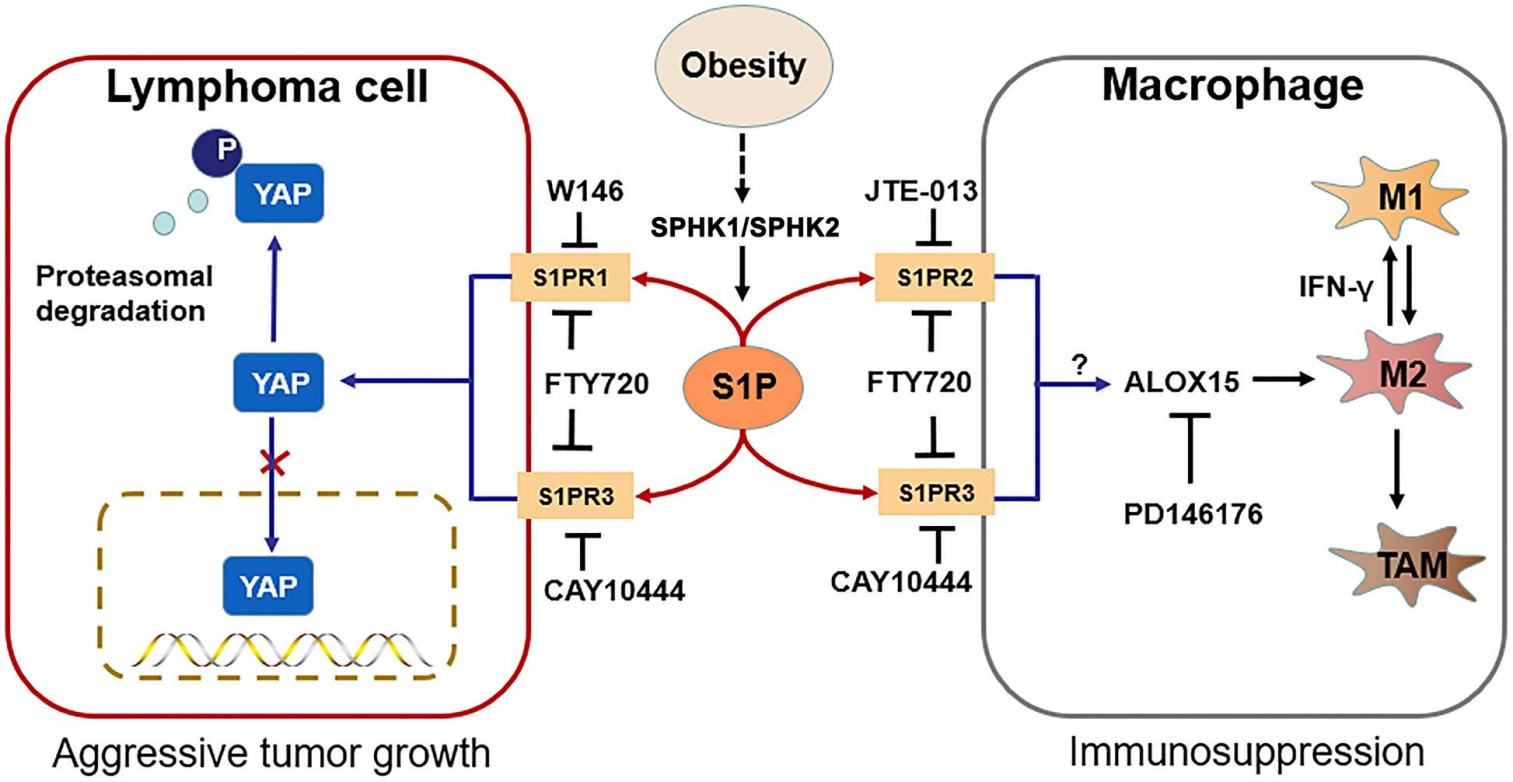

**Supplementary Information:**

The online version contains supplementary material available at 10.1186/s13046-022-02589-7.

## Highlights

• Obesity negatively impacted progression-free survival and overall survival in lymphoma patients while an aggressive tumor growth pattern was found in obesity-lymphoma mice.

• S1P-S1PR1/S1PR3-YAP signaling mediated lymphomagenesis contributing to tumor aggressive growth in obesity-lymphoma.

• S1P-ALOX15 signaling mediated TAMs contributing to immunosuppressive microenvironment in obesity-lymphoma.

• S1P-targeted therapy could be potentially effective and immune-enhancive against obesity-lymphomagenesis.

**Supplementary Information:**

The online version contains supplementary material available at 10.1186/s13046-022-02589-7.

## Introduction

According to World Health Organization (2016), more than 1.9 billion adults (18 years and older) were overweight (body mass index [BMI] value ≥25 kg/m^2^) in which over 650 million adults were obese (BMI value ≥30 kg/m^2^), the worldwide prevalence of obesity had been nearly tripled during past forty decades [1]. While overweight, obesity, and metabolic syndrome became epidemic conditions affecting 39, 13, and 20% of the population [2], individuals with obesity, in particular obesity with metabolic abnormalities, were found to be at an increased risk of cancers and with a greater mortality rate [3]. Accumulating epidemiological evidence indicates a causal link between obesity and cancer [4, 5] and metabolic disorder has been recognized as one of the most common features of various cancers [6, 7].

Lymphoma, a heterogeneous group of malignancies, comprises approximately 4.2% of new cases and 3.3% of deaths worldwide in 2019 [8]. Excess body weight has also been found to be associated with an increased risk of lymphomas, e.g., diffuse large B-cell lymphoma (DLBCL) [9, 10]. Some metabolic pathways are currently recognized in lymphomagenesis [11, 12], while the lipid metabolites such as sphingosine-1-phosphate (S1P) have been proposed to play an important role linking obesity and lymphomas [13]. S1P, a bioactive sphingolipid metabolite being produced by one of two sphingosine kinase isoforms (SPHK1 or SPHK2), can be released through transporters into the tumor microenvironment to influence tumor cells as well as immune cells, contributing to carcinogenesis [14]. Evidence supports the involvement of S1P signaling not only in solid tumors [14–18] but also in hematological malignancies [19]. In lymphomas, both increased S1P production and up-regulated S1P receptor 1 (S1PR1) are found and the S1P signaling is implicated through the S1P receptors [13, 20–22]. It has been demonstrated that S1P activates yes-associated protein (YAP) through S1P receptor 2 (S1PR2) in both begin and malignant cells [23, 24]. YAP is the core downstream effector of the Hippo pathway and mediates the expression of specific genes for cell proliferation. Whether the Hippo pathway may underpin the S1P-mediated induction of lymphoma cell proliferation remains unclear. The underlying mechanism of S1P/S1P receptor-YAP signaling in lymphomagenesis have not been elucidated.

In addition, it has been recognized that S1P and its receptors are involved in tumor/immune cell communication, as a novel modulator of immune plasticity in tumor microenvironment [25]. Macrophages, an abundant cell population in tumor microenvironment, possesses highly diverse phenotypic and functional heterogeneity by polarizing to either M1 or M2 sub-types, which can be educated by lymphoma cells to become immunosuppressive tumor assistants [26]. Tumor-associated macrophages (TAMs) are mainly M2 macrophages, while S1P can induce M2 polarization and facilitate macrophage chemotaxis and infiltration into the niche microenvironment [27, 28]. Dysregulation of lipid metabolism could render immunosuppressive microenvironment for the progression of lymphoma. In fact, the lipid metabolites, i.e., the arachidonic acid metabolites which are the major products of 12/15-lipoxygenases (ALOX15), have been turned out to be an important regulator of macrophage function [29]. ALOX15 exists in different mammalian isoenzymes designated together as 12/15-lipoxygenases since they have variable positional specificity to catalyze oxygenation at the 15-position and the 12-position of arachidonic acid. In human monocyte-derived macrophages, it has been reported that the formation of ALOX15 products and specialized pro-resolving mediators play critical roles during IL-4-induced M2 polarization [30]. However, the effect of S1P signaling on the S1P mediated TAMs is unknown.

Here, by using our established obesity-lymphoma mouse models as well as the lymphoma cell lines and a monocyte cell line, we revealed the potential mechanism of S1P/SPHK1 signaling in contribution to obesity-lymphomagenesis, in particular, the S1P-YAP axis in lymphoma cells and the S1P-mediated polarization of macrophages in the tumor microenvironment. Notably, we found that resveratrol showed the potential effects on not only blockage of S1P/SPHK1 signaling but also intervention of macrophage polarization in the tumor microenvironment of obesity-lymphoma. Combination of resveratrol and PD-L1 antibody enhanced the immune checkpoint blockade therapy in the obesity-lymphoma mice.

## Results

### Up-regulation of S1P/SPHK signaling in human lymphomas

**Fig. 1:**
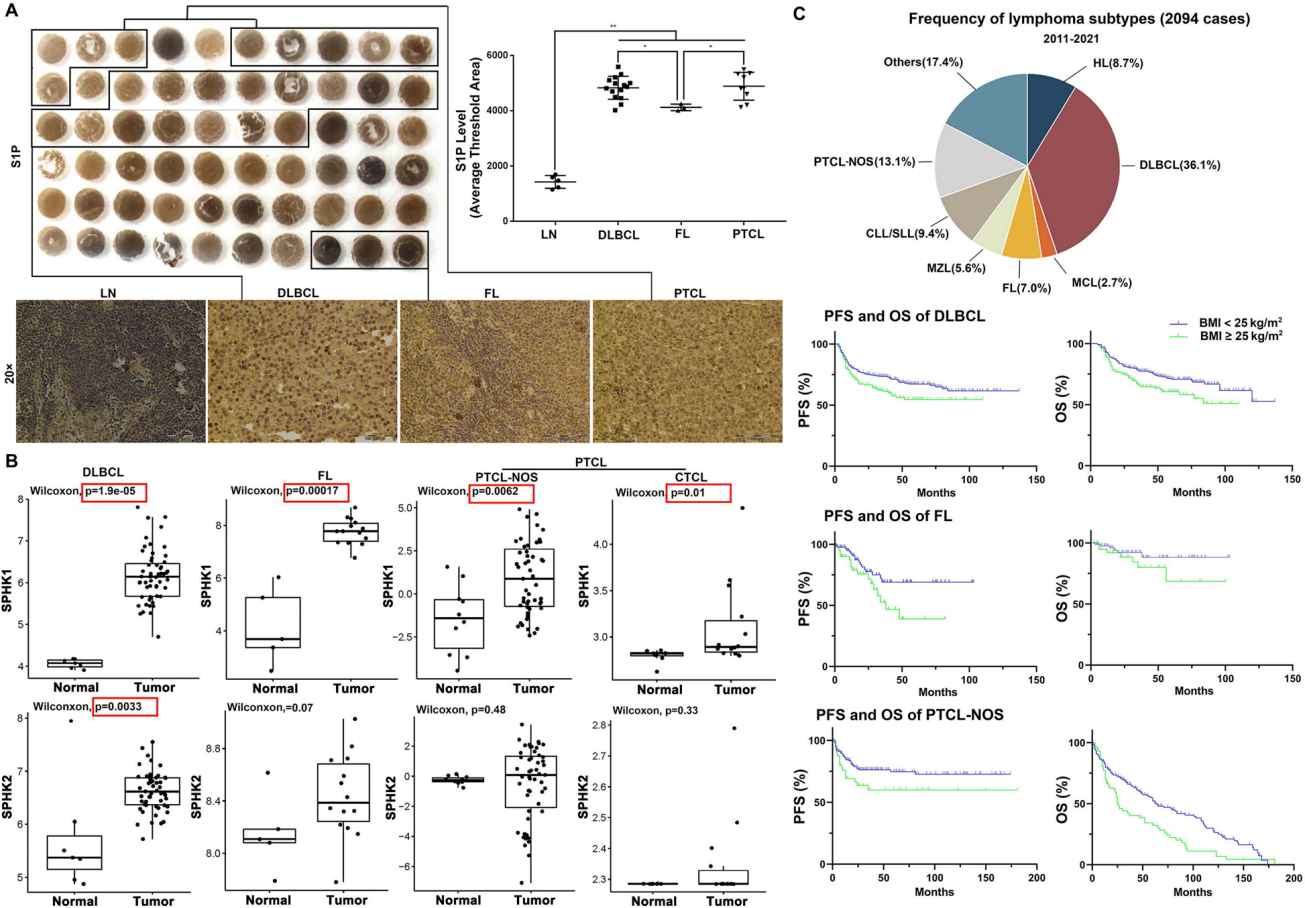
S1P/SPHK signaling in human lymphomas and PFS and OS in overweight lymphoma patients. **A** IHC staining of S1P was performed in the tissue array-samples from various human lymphomas and computer-imaging analysis indicated the S1P expression levels of three subtypes lymphomas, DLBCL, FL and PTCL, in comparison with the normal lymph node samples. **B** By using Gene Expression Omnibus (GEO) database, SPHK1 expression and SPHK2 expression were analyzed in DLBCL, FL and PTCL patients. **C** A cohort of 2094 lymphoma patients was recruited and PFS and OS in two groups (BMI ≥ 25 kg/m^2^ and BMI < 25 kg/m^2^) were analyzed in DLBCL, FL and PTCL-NOS patients. PFS: progression-free survival; OS; overall survival; DLBCL: Diffuse large B-cell lymphoma; FL: follicular lymphoma; PTCL: peripheral T-cell lymphoma; PTCL-NOS: peripheral T-cell lymphoma-not otherwise specified; CLL/SLL: Chronic lymphocytic leukemia/Small lymphocytic lymphoma; HL: Hodgkin’s Lymphoma; MZL: Marginal zone lymphoma; MCL: Mantle cell lymphoma; CTCL: Cutaneous T-cell lymphoma. *, *P* < 0.05; **, *P* < 0.01

Although existing data indicate the carcinogenetic role of S1P/SPHK signaling, it is not well documented in lymphomas. An IHC study was performed to determine the levels of S1P in the tissue array-samples from various human lymphomas (detailed information in supplementary file Table 3). By computer-imaging analysis, significantly increased S1P levels were detected in the tissues from DLBCL, follicular lymphoma (FL), and peripheral T-cell lymphoma (PTCL) (Fig. 1A). Using a web-based database, Gene Expression Omnibus (GEO), the high throughput gene expression was profiled for SPHK1 and SPHK2 in the lymphomas (DLBCL, FL, and PTCL) and the respective normal controls. Significantly up-regulated expression of SPHK1 was found in DLBCL (*n* = 55) versus centroblast B-cells from huma tonsils (*n* = 7), in FL (*n* = 14) versus normal B-cells (n = 5), and in PTCL-NOS (*n* = 68) versus normal T cells (*n* = 10). Significantly up-regulated expression of SPHK2 were also detected in DLBCL but not in FL and PTCL in comparison with their respective normal controls (Fig. 1B). The impact of obesity on lymphomas was further investigated, retrospective analysis in a cohort of 2094 lymphoma patients indicated that overweight (BMI ≥ 25 kg/m^2^) had a negative impact on progression-free survival (PFS) and overall survival (OS) in the DLBCL, FL, and PTCL-NOS patients (Fig. 1C). Taken together, the increased S1P levels, up-regulated sphingosine kinases, and overweight negatively impacting PFS and OS in lymphoma patients evoked the interest to explore the obesity-associated S1P/SPHK signaling in lymphomagenesis.

### Aggressive tumor growth pattern in obesity-lymphoma mice

**Fig. 2:**
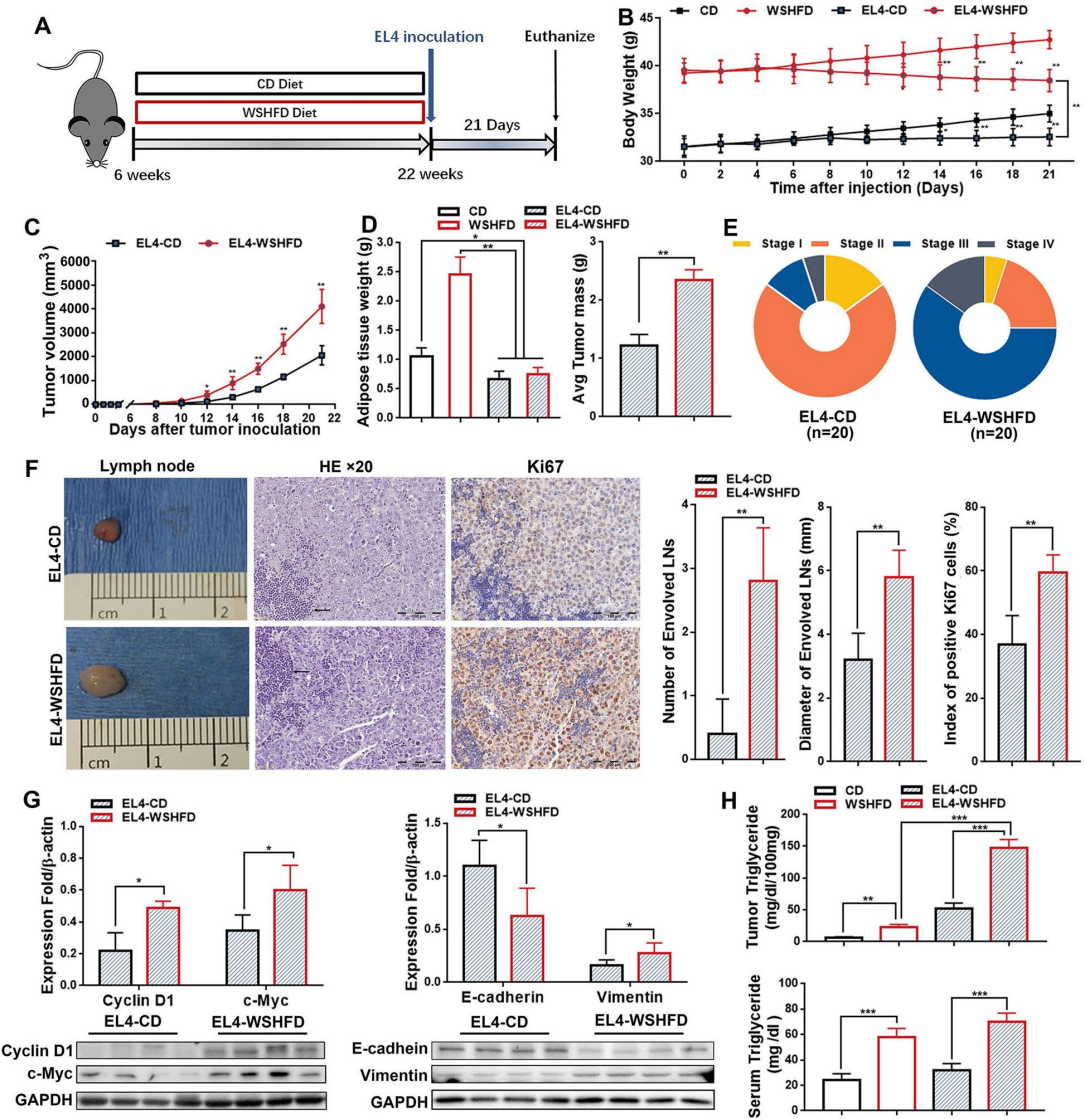
Tumor growth in obesity-lymphoma mice. **A** Schematic diagram for establishing obese mouse model by WSHFD and establishing obesity-lymphoma model by EL4 cells subcutaneous xenograft in obese mice. **B** The body weight change after xenograft in EL4-WSHFD mice and EL4-CD mice, as well as the non-tumor controls. **C** The changes of tumor volume after lymphoma cell xenograft in EL4-WSHFD mice and EL4-CD mice. **D** The adipose tissue weight and tumor weight in EL4-WSHFD mice and EL4-CD mice measured at sacrifice. **E** The lymphoma staging according to Ann Arbor staging classification in EL4-WSHFD mice and EL4-CD mice. **F** Representative images of gross anatomy of harvested lymph nodes, histology by H&E, and Ki-67 staining by IHC in the tumor tissues from EL4-WSHFD mice and EL4-CD mice. For the histological details in H&E staining, the extensively distributed lymphoma cells were showing at least three to five times the size of the normal lymphocytes (arrow). The lymphoma cells had round nuclear outlines, vesicular chromatin, and single to multiple prominent nucleoli. For the Ki-67 staining, most Ki-67 positive cells are lymphoma cells (brown color). **G** Western blot analysis for the protein levels of cyclin D1, c-Myc, E-cadherin, and Vimentin in the tumor tissues from EL4-WSHFD mice and EL4-CD mice. **H** Triglyceride levels in the serum and tumor tissue from EL4-WSHFD mice and EL4-CD mice, as well as the non-tumor controls. *, *P* < 0.05; **, *P* < 0.01 ***, *P* < 0.001

High fat consumption is closely related to the development of obesity [31], while high fat with high fructose increases the similarity to typical Western diet style which recapitulate the pathogenesis of lipid metabolic disorder and supposedly potentiates its metabolically deleterious effects [32]. Using an animal model, the tumor growth in obesity was further evaluated. Six-week-old C57BL/6 mice were fed with the Western style high fat diet (WSHFD, 35% w/w fat and 26% w/w fructose) to establish an optimal “metabolic disorder window” for lymphoma cells inoculation. After 16 weeks WSHFD-feeding, significantly increased serum triglycerides, body weight, and adipose tissue weight along with the biomarkers of metabolic disorders (increased phosphorylation of hormone-sensitive lipase, adipocyte size, and inflammatory infiltration, but decreased insulin receptor substrate 1) were found in the WSHFD mice, with statistical significances compared to the control diet (CD) feeding (Fig. S1). The WSHFD-obese mice as well as the CD-control mice were injected with EL4 cells subcutaneously (s.c.) at 5 × 10^6 cells for tumor xenografts (Fig. 2A). Although the body weights of tumor-burdened mice were significantly decreased in comparison to their respective non-xenograft controls, the body weights of EL4-WSHFD mice were much higher than the EL4-CD mice (Fig. 2B). Significantly increased tumor volume was found at day 12 and continued to increase till sacrifice in the EL4-WSHFD mice compared to the EL4-CD mice (Fig. 2C). After sacrifice, the adipose tissues were weighted and found significantly decreased in the tumor-burdened mice in comparison to the non-xenograft mice (Fig. 2D), which was consistent with the decreased body weight. In comparison with the EL4-CD mice, the aggressive tumor growth pattern in the EL4-WSHFD mice was evident as the significant increased weight of tumor mass and the advanced stage (Fig. S2 and Fig. 2E), extensive tumor invasion of the draining lymph nodes (LNs), and significantly increased proliferation by Ki-67 staining in the LN tissues which infiltrated with tumor cells (Fig. 2F). The cytologic characteristics of the infiltrated lymphoma cells by H&E staining showed non-cleaved and plasmacytoid, at least two to three times in size versus normal lymphocyte, with round nuclear outlines, vesicular chromatin, and single to multiple prominent nucleoli (Fig. 2F). Further evidence of aggressive tumor growth of the WSHFD-EL4 mice was showed by Western blot analysis of the protein levels of cyclin D1, c-Myc, and E-cadherin and Vimentin for epithelial–mesenchymal transition (EMT) in comparison with the CD-EL4 mice (Fig. 2G). Interestingly, significant increases of triglyceride levels in tumor tissues were found in the WSHFD-EL4 mice, compared to either the non-xenograft mice with WSHFD-feeding or the CD-EL4 lymphoma mice (Fig. 2H). As cancer cells are much plastic and flexible in metabolism to support their rapid growth, we therefore further study lipid metabolic remodeling, particularly the S1P metabolism, in the WSHFD-EL4 lymphoma mice.

### Increased S1P synthesis and up-regulated S1PR in obesity-lymphoma mice

**Fig. 3:**
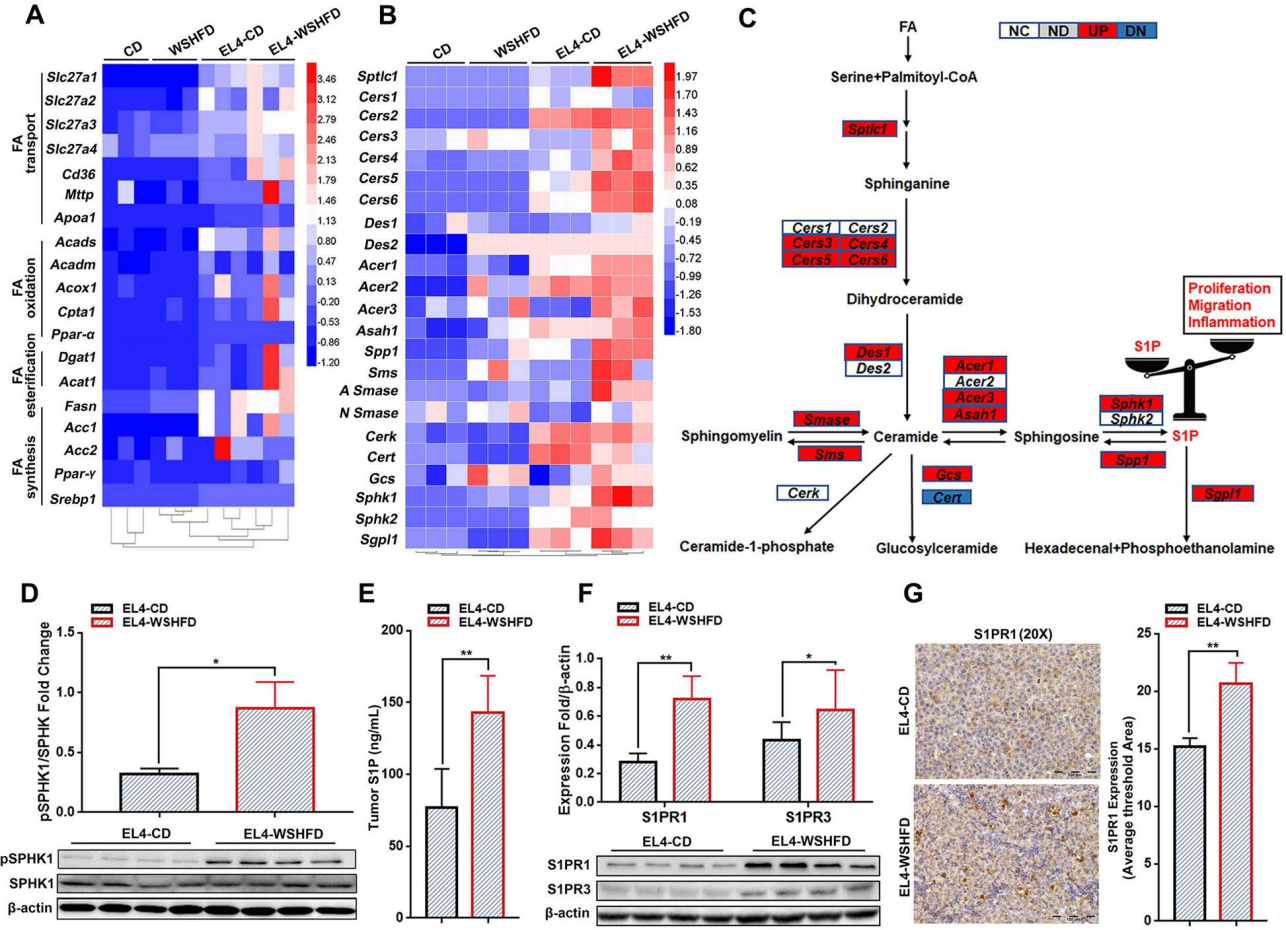
Lipid metabolism, S1P synthesis and S1P receptors in obesity-lymphoma mice. **A** Heat map of the key FFAs metabolic enzymes including FA transport (Slc27a1, Slc27a2 Slc27a3, Slc27a4, and CD36), FA oxidation (Acads, Acadm, Acox1, Cpt1a, and PPAR-α), export (Mttp and Apoa1), esterification (Dgat1 and Acat1) and FA synthesis (FASN, ACC1, ACC2, PPAR-γ and Srebp1) by q-PCR analysis in the tumor tissues from EL4-WSHFD mice and EL4-CD mice, as well as the non-tumor controls. **B** Heat map of the enzymes for S1P synthesis by q-PCR analysis in the tumor tissues from EL4-WSHFD mice and EL4-CD mice, as well as the non-tumor controls. **C** Schematic diagram of S1P biosynthetic cascade enzymes for the fold changes of WSHFD-EL4 mice versus CD-EL4 mice. ND, no detection; NC, no change; Red, > 2-fold up-regulated; Blue, < 2-fold down-regulated. **D** Western blot analysis for the protein levels of SPHK1 and phosphorylated SPHK1 in the tumor tissues from EL4-WSHFD mice and EL4-CD mice. **E** The levels of S1P production in the tumor tissues from EL4-WSHFD mice and EL4-CD mice. **F** Western blot analysis for the protein levels of the S1P receptors, S1PR1 and S1PR3. **G** Representative images of IHC staining for S1PR1 and the computer-imaging analysis of S1P expression levels in the tumor tissues from EL4-WSHFD mice and EL4-CD mice. X20, 200-fold magnification. *, *P* < 0.05; **, *P* < 0.01

To examine the metabolic remodeling in the WSHFD-feeding mice, VO_2_ (ml/kg/hour) and VCO_2_ (ml/kg/hour) was monitored using the metabolic cages. Whole-body calorimetry showed significantly decreased levels of respiratory exchange ratio (RER) and energy expenditure in the WSHFD-feeding mice compared to the CD-feeding controls (Fig. S3), suggesting an increased use of lipid fuels for energy in the WSHFD induced obese mice. Given that the aberrant lipid metabolism can affect a variety of carcinogenetic pathways, a qPCR assay was performed to profile gene expression of lipid metabolic enzymes. Up-regulated mRNA levels of the enzymes for fatty acid (FA) synthesis, FA esterification, FA oxidation, and FA transport, were found in the tumor tissues from EL4-lymphoma mice, especially from the WSHFD-EL4 mice (Fig. 3A), indicating that the aberrant lipid metabolism linked to aggressive tumor growth in obesity-lymphoma mice. Further analysis for S1P cascade synthetic enzymes (including *Sptlc1, Cers* [1–6]*, Des* [1, 2]*, Acer* [1–3]*, Asah1, Smase, Sms1, Cerk, Cert, Gcs, SphK1, SphK2, Spp1,* and *Sgpl1*) showed that most enzymes were upregulated in the tumor tissues of EL4-WSHFD mice (Fig. 3B) and the result of fold changes (WSHFD-EL4 vs CD-EL4) revealed upregulation of those enzymes for the biosynthesis of sphinganine, sphingosine, ceramide and S1P(Fig. 3C). Western blotting for phosphorylated SPHK1 and ELISA for S1P further confirmed that S1P production was significantly increased in the tumor tissues of EL4-WSHFD mice (Fig. 3D-E), indicating that S1P could play a key role contributing to the malignant events such as proliferation and migration in the EL4-WSHFD mice. S1P exerts its bioactivities such as proliferation and migration via binding to a family of G protein-coupled receptors, known as S1PR1–5 [33]. In the tumor tissues of EL4-WSHFD mice, significantly increased protein levels of S1PR1 and S1PR3 by Western blotting were found (Fig. 3F) and the finding was consistent with previous studies in which S1PR1 and S1PR3 were reported as overexpression of the two most important signaling molecules involved in the lymphomagenesis [22, 34]. IHC of S1PR1 showed the positive staining in most lymphoma cells (Fig. 3G). All the data indicated that increased S1P production and up-regulated S1P receptors played important roles contributing to the lymphomagenesis in obesity-lymphoma mice.

### S1P-S1PR1/S1PR3-YAP signaling mediated lymphomagenesis

**Fig. 4:**
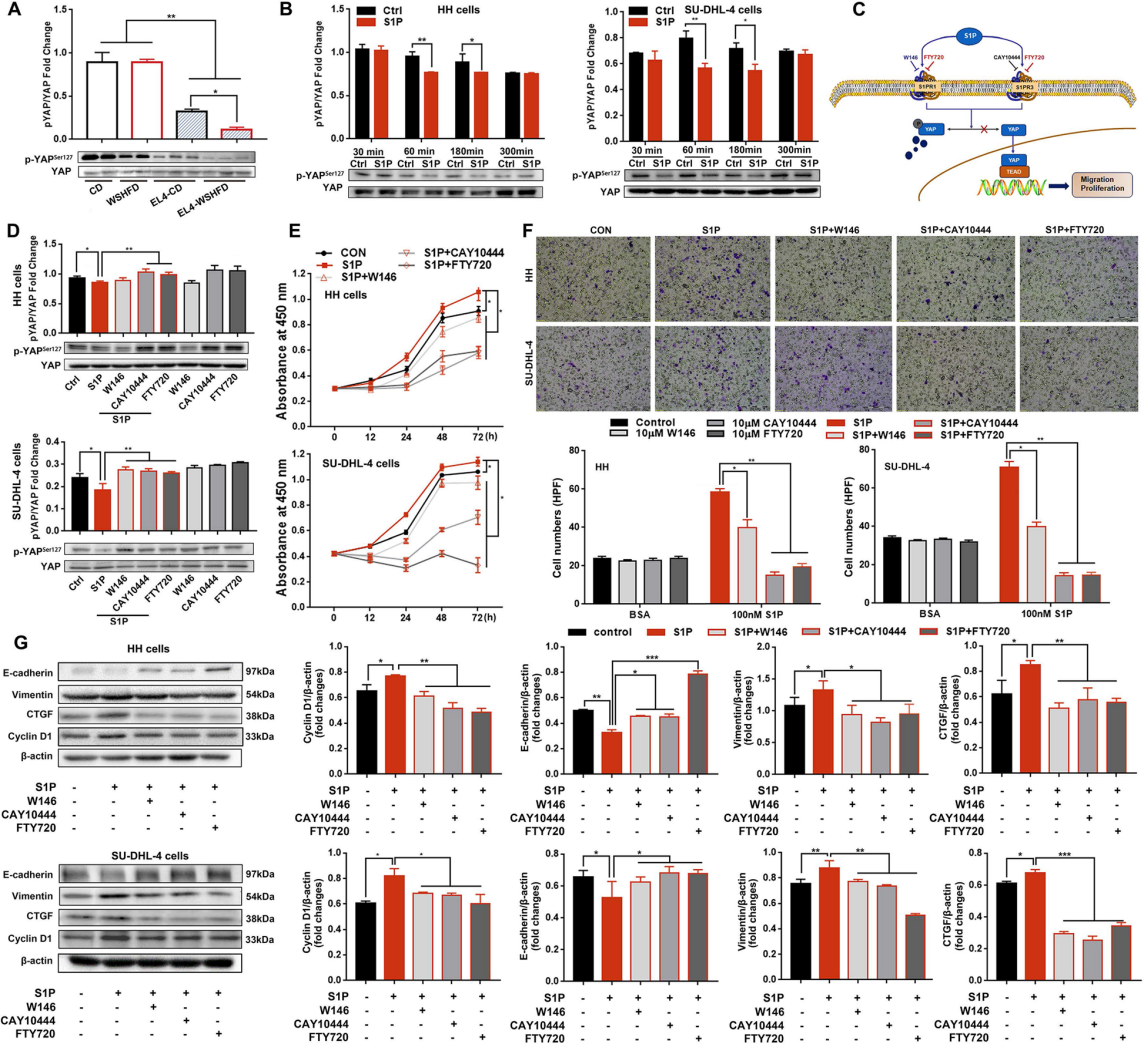
The S1P-S1PR1/S1PR3-YAP signaling. **A**, **B** Western blot analysis for the protein levels of phosphorylated YAP in the tumor tissues from mice and in the lymphoma cells treated with S1P. **C** Schematic diagram of S1P/S1PR1 mediated YAP signals. **D** Western blot analysis for the protein levels of phosphorylated YAP in the lymphoma cells treated with S1P and inhibitors of S1P receptors. **E**, **F** A XTT cell viability assay and a trans-well assay as well as Wester blotting for cell proliferation and migration of the lymphoma cells treated with S1P and inhibitors of S1P receptors. **G** Western blot analysis for the protein levels of E-cadherin and Vimentin for EMT, CTGF for YAP targeting gene, and cyclin D1 for cell cycle progression in the lymphoma cells treated with S1P and inhibitors of S1P receptors. *, *P* < 0.05; **, *P* < 0.01; ***, *P* < 0.001

The S1P-S1P receptors mediated YAP activation has been reported previously in various malignances [24, 35, 36] but not in lymphomas. In current study, significantly decreased protein level of phosphorylated YAP by Western blotting was found in the tumor tissue of EL4-lymphoma mice (Fig. 4A). This result encouraged us to further investigate the potential mechanism of YAP signaling contributing to lymphoma. To investigate mechanism of S1P/S1P receptor-YAP signaling in lymphomagenesis, two human lymphoma cell lines (HH, T cell lymphoma cells and SU-DHL-4, a B cell lymphoma) along with S1PR inhibitors were used. The S1P mediated down-regulation of phosphorylation of YAP was found in both HH cells and SU-DHL-4 cells (Fig. 4B). Gene expressions of S1PR1, S1PR2 and S1PR3 were detected by qPCR assay, but S1PR4 and S1PR5 were undetectable. By Western blotting, high protein levels of S1PR1 and S1PR3, and low protein levels of S1PR2 were detected but not S1PR4 and S1PR5, which was consistent with the qPCR results (Fig. S4B). Three S1PR inhibitors (W146 for S1PR1, CAY10444 for S1PR3, and FTY720 for S1PR1–5) were selected to demonstrate the hypothesis that S1P signaling could mediate the HIPPO pathway in lymphoma cells (Fig. 4C). The results indicated that blockage of S1PR1 and/or S1PR3 significantly attenuated the S1P-induced decreases of YAP phosphorylation in both HH cells and SU-DHL-4 cells (Fig. 4D). To investigate the carcinogenetic cellular events, a XTT cell viability assay and a trans-well assay were performed to investigate cell proliferation and migration. Consistently, blockage of S1PR1 and/or S1PR3 significantly inhibited the S1P induced cell proliferation and migration (Fig. 4E-F). Cyclin D1 had been reported as the biomarker for cell cycle arrest in both HH cells and SU-DHL-4 cells [37, 38]. Western blotting analysis was therefore performed to determine if blockage of S1PR1 and/or S1PR3 could affect the cell cycle arrest and EMT events further, down regulated protein level of cyclin D1 and attenuated the alteration of E-cadherin/Vimentin was found in both HH cells and SU-DHL-4 cells with S1P treatment (Fig. 4G). Blockage of S1PR1 and/or S1PR3 also significantly down-regulated connective tissue growth factor (CTGF), a YAP transcriptional targeting gene, demonstrating that the S1P-S1PR1/S1PR3-YAP axis contributed to the lymphomagenesis.

### Macrophage polarization in obesity-lymphoma mice

**Fig. 5:**
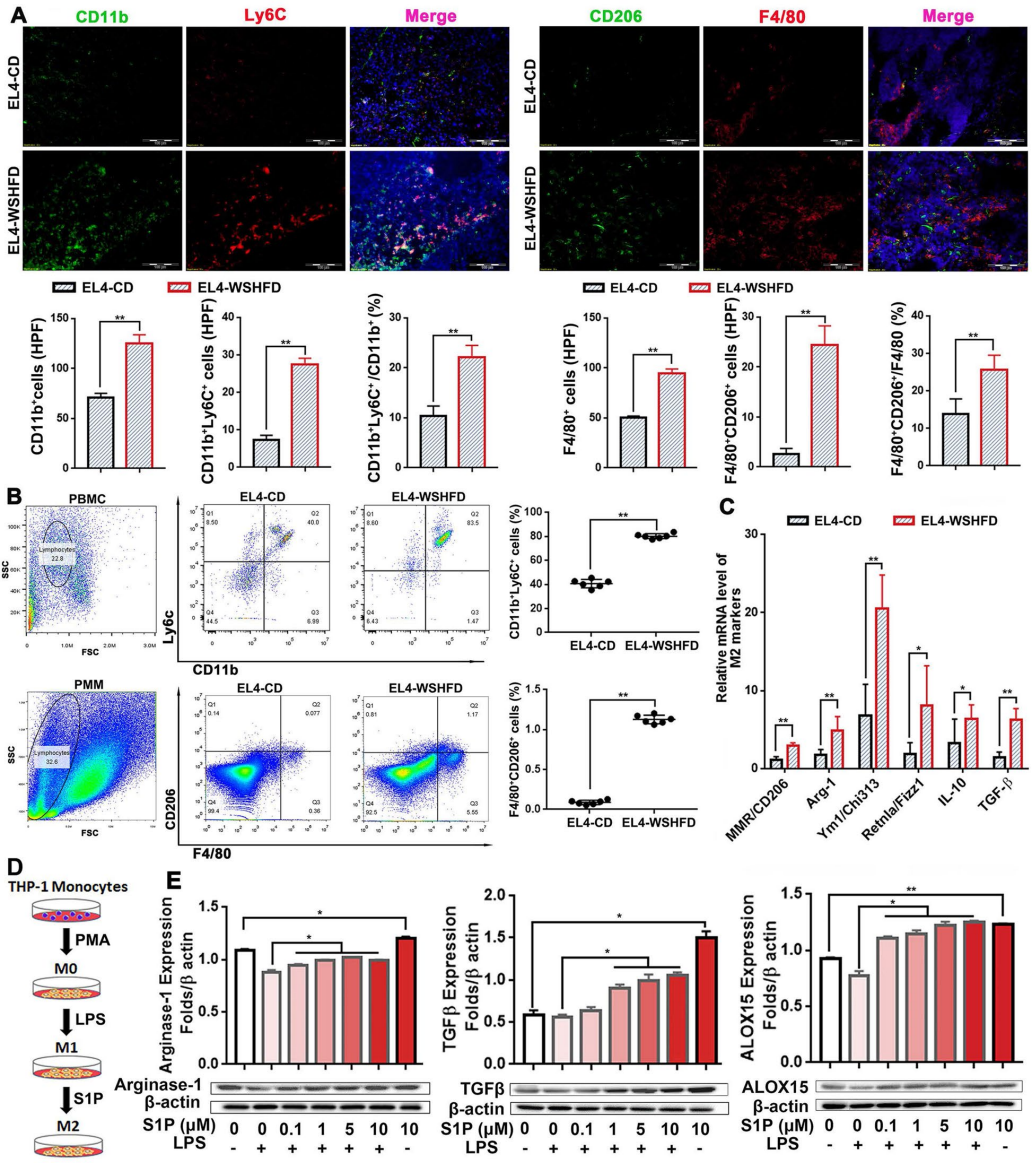
TAMs and macrophage polarization in obesity-lymphoma mice. **A** In the tissues of the tumor invaded lymph node from xenograft model of EL4-WSHFD mice and EL4-CD mice, immunofluorescent staining was performed using the antibodies of anti-CD11b and anti-Ly6C as well as the antibodies of anti-CD206 and anti-F4/80 to detect the M-MDSC derived macrophages. Green: positive staining for CD11b or CD206; red: positive staining for Ly6C or F4/80; blue: positive DAPI (4′,6-diamidino-2-phenylindole) staining to detect the nuclei as a counterstain. **B** Flow Cytometry analysis to detect CD11b^+^Ly6C^+^ cells and F4/80^+^CD206^+^ cells in the collected PBMC and PMM from peritoneal injection model of EL4-WSHFD mice and EL4-CD mice. **C** q-PCR analysis of M2 phenotype in the collected PMM from peritoneal injection model of EL4-WSHFD mice and EL4-CD mice. **D** Schematic diagram of PMA induced M0 phenotype, LPS induced M1 phenotype, and S1P induced M2 phenotype in THP-1 monocytes. **E** Western blot analysis for the protein levels of arginase-1, TGFβ, and ALOX15 in the PMA induced M0 THP-1 monocytes treated with LPS and/or S1P. HPF: high-power field; PMA: phorbol 12-myristate 13-acetate; PMM: peritoneal monocytes/macrophages. Scale bar = 100 μm. *, *p* < 0.05; **, *p* < 0.01

The cellular interaction between lymphoma cells and aberrant myeloid cells has been suggested to involve in lymphomagenesis during ensuing lymphoma development [39]. The remarkable players in tumor-dependent immune dysfunction are myeloid-derived suppressor cells (MDSCs), including two major subsets: polymorphonuclear (PMN)-MDSC and monocytic (M)-MDSC [40]. In mice, MDSCs phenotypically were defined as cells expressing markers, PMN-MDSC (CD11b^+^Ly6G^+^Ly6C^lo^) and M-MDSC (CD11b^+^Ly6G^−^Ly6C^hi^), while tumor-associated macrophages (TAMs) can be distinguished from M-MDSCs by decreased expression of Ly6C but increased expression of F4/80 and CD206 [41]. In the EL4-lymphoma mice, fluorescent staining showed significantly increased CD11b^+^Ly6C^+^ cells and F4/80^+^CD206^+^ cells in the tumor invaded lymph nodes, compared to that in the EL4-CD mice (Fig. 5A). These sub-populations of myeloid cells in mouse lymph nodes suggested that the infiltrated monocyte/macrophage cell lineage most likely contributed to TAMs. To study the aberrant myeloid cells in the tumor micro-milieu, we established an EL4-lymphoma model by peritoneal injection with EL4 cells in WSHFD-feeding mice as well as CD-feeding controls. One week after EL4 cells injection, mouse peripheral blood mononuclear cell (PBMC) and peritoneal monocyte/macrophage (PMM) were collected for Flow cytometry analysis. Significantly increased subpopulations of CD11b^+^Ly6C^+^ cells and F4/80^+^CD206^+^ cells but decreased CD8^+^ cells were found in either the PBMCs or the isolated PMM from WSHFD-feeding mice compared to that in CD-feeding mice (Fig. 5B, Fig. S5). Further analysis by qPCR in the isolated PMM indicated that the biomarkers of M2 phenotype were significantly up-regulated in WSHFD-feeding mice compared to the CD-feeding mice (Fig. 5C). To determine that S1P microenvironment could influence M2-polarization of macrophages, an in vitro study was performed using THP-1 cells, a human monocyte cell line. The THP-1 cells were firstly treated with 10 μg/ml phorbol 12-myristate 13-acetate (PMA) for 48 hours to induce the M0 phenotype, then the M0 THP-1 cells were treated with LPS and/or S1P. With LPS treatment, the M0 THP-1 cells showed M1-polarizaytion as evident by the down-regulated arginase-1 and TGF-β, however S1P treatment attenuated the LPS induced down-regulated arginase-1 and TGF-β in a dose-dependent manner, indicating that S1P could alter the macrophage polarization towards M2 phenotype (Fig. 5D-E). In tumor microenvironment, arachidonic acid as an active lipid metabolite plays a key role to enhance cancer-related inflammation which is associated with the tumor-induced immune suppression [42, 43]. ALOX15, a well-studied member of LOX family for converting arachidonic acid, has been reported to be selectively potentiated in macrophages and play a key role for alternatively activated macrophages (AAMs), while expression of ALOX15 is referred as M2 macrophages [44]. In the S1P treated THP-1 cells, we found significantly increased protein level of ALOX15 in association with the M2-polarizaytion (Fig. 5D). Taken together, the data indicated that WSHFD-induced obesity caused active lipid metabolites and the S1P mediated ALOX15 signaling could be the potential mechanism for macrophage polarization towards M2 phenotype and TMAs, while recruitment of myeloid cells might play a key role contributing to immunosuppression.

### S1P-ALOX15 signaling mediated macrophage polarization towards TAMs

**Fig. 6:**
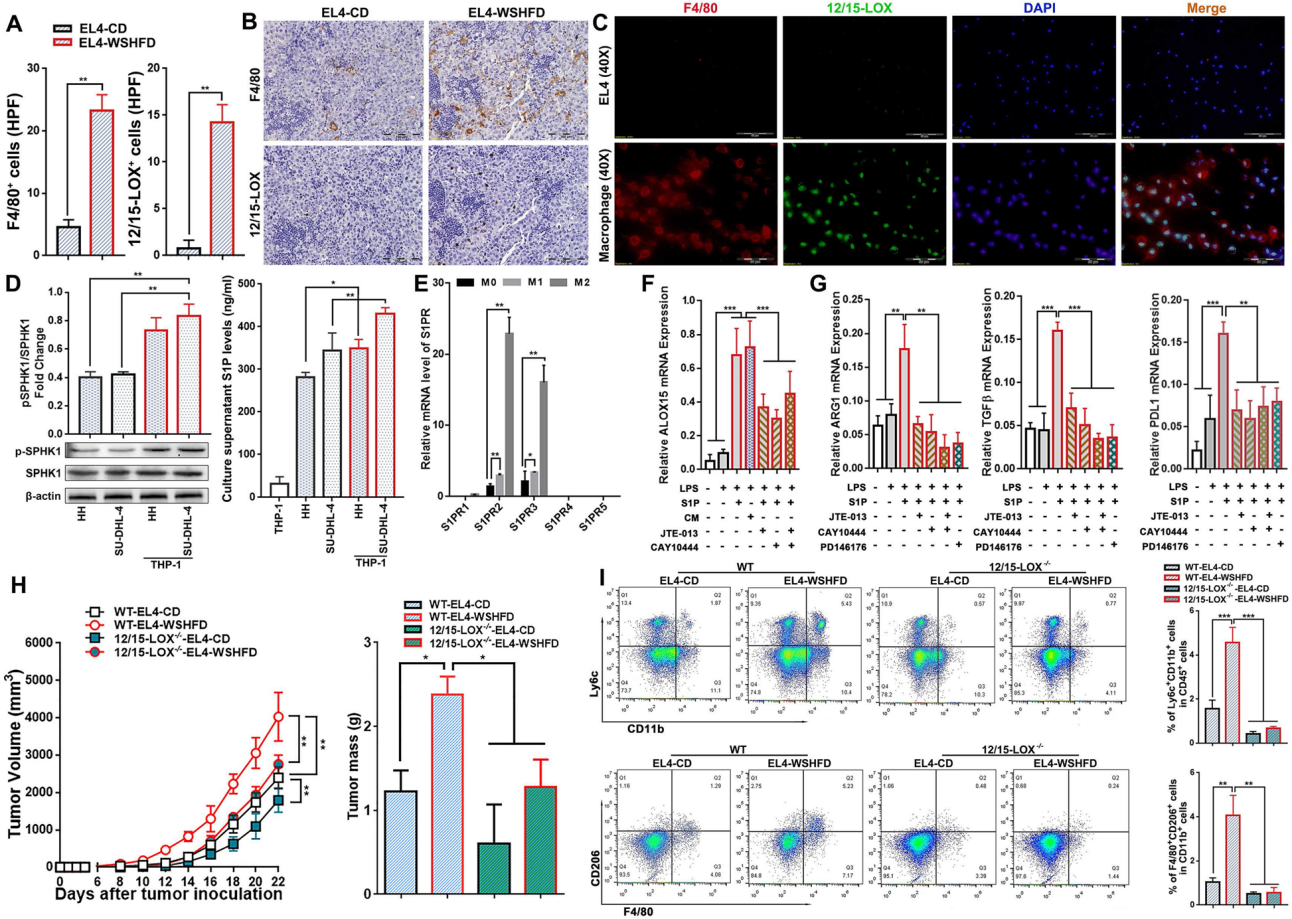
S1P-ALOX15 signaling mediated TAMs. **A** Computer-imaging analysis for the 12/15-LOX positive cells and F4/80 positive cells detected by IHC staining in the tumor tissues from xenograft model of EL4-WSHFD mice and EL4-CD mice. **B** Representative images of IHC in serial sections from the same paraffin tissue block to detect 12/15-LOX positive cells and F4/80 positive cells from the xenograft model of EL4-WSHFD mice and EL4-CD mice. **C** Dual immunofluorescent staining using the antibodies of anti-F4/80 and anti-12/15-LOX to confirm the co-localization of 12/15-LOX positive cells and F4/80 positive cells in isolated macrophages from EL4-WSHFD mice in comparison with EL4 cells. **D** The protein levels of phosphorylated SPHK1 and SPHK1 by Western blot in lymphoma cells (HH and SU-DHL-4) co-cultured with THP-1 cells and the S1P levels by the enzyme-linked immunosorbent assay (ELISA) in the supernatant of co-culture medium. **E** The mRNA levels of S1P receptor (1–5) by q-PCR in the THP-1 monocytes, M0 phenotype induced by PMA, M1 phenotype induced by LPS, and M1 phenotype induced by S1P. **F** The mRNA levels of ALOX15 in the THP-1 monocytes treated with LPS, S1P, co-culture medium, and inhibitors of S1P receptors. **G** The mRNA levels of arginase-1, TGFβ, and PD-L1 in the THP-1 monocytes treated with LPS, S1P, inhibitors of S1P receptors, and inhibitors of ALOX15. **H** The tumor volume changes after xenograft and tumor mass weight measured at sacrifice in 12/15-LOX^−/−^-EL4-WSHFD mice and WT-EL4-CD mice. **I:** Flow Cytometry analysis to detect the subpopulations of F4/80^+^CD11b^+^ cells and F4/80^+^CD206^+^ cells for potential TAMs in the collected the PMM from the peritoneal injection model of 12/15-LOX^−/−^-EL4-WSHFD mice and WT-EL4-CD mice. CM: co-culture medium; PMM: peritoneal monocytes/macrophages; HPF: high-power field. Scale bar = 100 μm in IHC staining. Scale bar = 50 μm in dual immunofluorescent staining. *, *p* < 0.05; **, *p* < 0.01; ***, *p* < 0.001

To evaluate the ALOX15 signaling in human lymphoma, gene expression of ALOX15 was analyzed in lymphoma patients using the GEO database. The results indicated that the mRNA levels of 12/15-LOX were significantly upregulated in DLBCL patients (*n* = 55) and PTCL patients (*n* = 68) in comparison with their respective B cell controls (Centroblast B-cell donors, *n* = 7) and T cell controls (Human T cell donors, *n* = 10) (Fig. S6B). Using the Tumor Immune Estimation Resource (TIMER) database, the survival rate was analyzed in the DLBCL patients. By splitting 25% of the patients with high level of macrophages and 25% with low level of macrophages, the KM curve showed a poor outcome for the patient with high level of macrophages (Fig. S6C). The results from lymphoma patients encouraged us to further investigate relationship between ALOX15 and macrophage in the animals. Significantly increased 12/15-LOX (ALOX15) positive cells and F4/80 positive cells were detected by IHC staining in EL4-WSHFD mice compared to EL4-CD mice (Fig. 6A). In the serial sections from same paraffin block, the positive cells of 12/15-LOX and F4/80 were distributed into the same regions (Fig. 6B), implying that macrophages might express high levels of 12/15-LOX in EL4-WSHFD mice. By dual fluorescent staining, we further investigated the co-expression of F4/80 and 12/15-LOX in the isolated macrophages from EL4-WSHFD mice and found that F4/80 localized mostly on the cell membrane while 12/15-LOX localized mainly in nucleolus, however, 12/15-LOX was not detected in the EL4 lymphoma cells (Fig. 6C), which was consistent to previous report that lymphoma cells did not express ALOX15 [45]. This result revealed that there could be a potential mechanism of crosstalk between lymphoma cells and macrophages. To explore the potential mechanism, an in vitro study was therefore performed using the PMA induced M0 THP-1 cells to co-culture with HH cells or SU-DHL-4 cells. Significantly increased phosphorylated SPHK1 in the co-cultured THP-1 cells and significantly increased S1P production in the co-culture medium were found but very low level of S1P was detected in the medium of THP-1 cells without co-culture (Fig. 6D), indicating that most S1P production was from the co-cultured lymphoma cells (HH cells or SU-DHL-4 cells). S1P receptors were further determined in M0-, M1- and M2-THP-1 cells by qPCR analysis. Significantly upregulated S1PR2 and S1PR3 were found in the M2-THP-1 cells (Fig. 6E), suggesting that S1P/S1P2/ S1PR3 signaling might play an important role to mediate macrophage’s 15-lipoxygenase expression and induce TAMs. To test this hypothesis, the LPS induced M1-THP-1 cells were treated with S1P or the co-culture medium of HH cells. Significantly upregulated ALOX15 were found by the treatments of either co-culture medium or S1P, while blockage of S1PR2 and S1PR3 significantly attenuated ALOX15 protein level (Fig. 6F), indicating the existence of S1P-ALOX15 axis via crosstalk between HH cells and THP-1 cells. To study whether the upregulated ALOX15 could be associated with the M2-polarized macrophages towards the TMAs, arginase-1, TGF-β, and PD-L1 which were reported as the TAM makers [46] were further determined in the S1P-challenged THP-1 cells. As expected, the S1P induced up-regulation of arginase-1, TGF-β and PD-L1 were significantly attenuated by either S1PR inhibitors or ALOX15 inhibitor (Fig. 6G). To validate the macrophages-ALOX15 signaling mediated lymphomagenesis in vivo, we established two EL4-lymphoma models by either subcutaneous xenograft or peritoneal injection in 12/15-LOX knockout (12/15-LOX^−/−^) mice as well as wild-type (WT) controls. In the subcutaneous xenograft model, significantly decreased tumor volume/weight was found in the 12/15-LOX^−/−^ mice in comparison with the WT controls (Fig. 6H). Significantly decreased CD11b^+^Ly6C^+^ cells and F4/80^+^CD206^+^ cells by fluorescent staining were found in the tissues of tumor invaded lymph node from 12/15-LOX knockout mice in comparison to WT mice (Fig. S7), suggesting that less-infiltration of M2 macrophages in the tumor microenvironment was associated with the decreased tumor weight. Flow Cytometry analysis was therefore performed in the isolated PMM from the peritoneal injection model of EL4-WSHFD mice, and the results showed significantly decreased CD11b^+^Ly6C^+^ cells and F4/80^+^CD206^+^ cells in the 12/15-LOX^−/−^ mice compared to the WT mice (Fig. 6I), confirming the finding from xenograft model. Taken together, the S1P-ALOX15 signaling mediated macrophage polarization towards TAMs could be a potential mechanism rendering the immunosuppressive microenvironment in obesity-lymphoma.

### Combination of resveratrol and anti-PD-L1 antibody suppresses lymphomagenesis

**Fig. 7:**
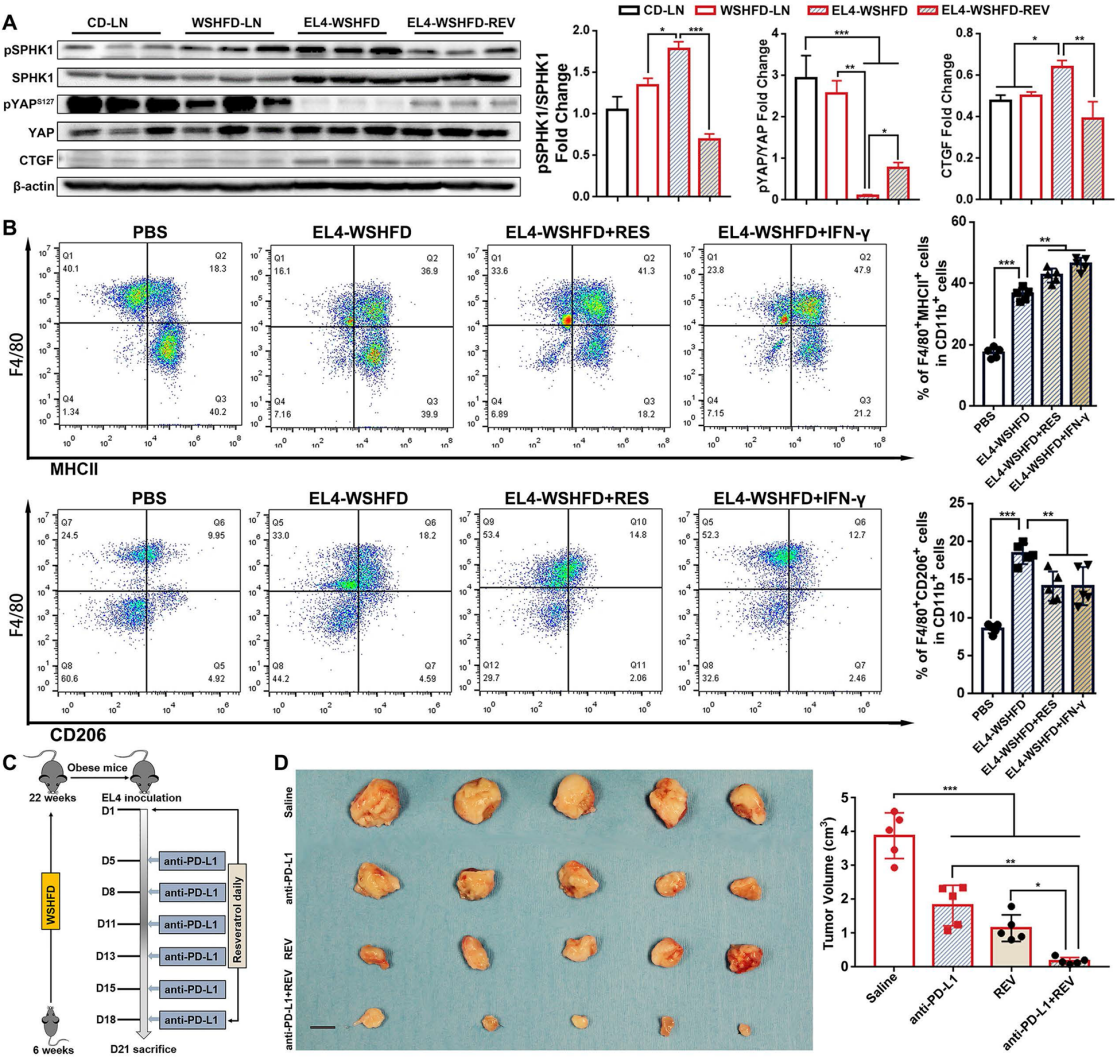
Therapy using resveratrol and anti-PD-L1 in obesity-lymphoma mice. **A** Protein levels of phosphorylated-SPHK1, SPHK1, phosphorylated-YAP, YAP, and CTGF in tumor invaded LN tissues from the xenograft model of resveratrol treated EL4-lymphoma mice and untreated EL4-lymphoma mice, in comparison with the lymph node from the non-lymphoma mice. **B** Flow Cytometry analysis to detect F4/80^+^MHCII^+^ cells and F4/80^+^CD206^+^ cells in the collected PMM from peritoneal injection model of resveratrol and IFN-γ treated EL4-lymphoma mice, in comparison with untreated EL4-lymphoma mice and the PBS injection non-tumor controls. **C** Schematic diagram of resveratrol and anti-PD-L1 treatment in xenograft model of EL4-lymphoma. **D** Gross anatomy of tumor mass, and tumor volume in 4 groups of the EL4-WSHFD mice. *, *P* < 0.05; **, *P* < 0.01. ***, *P* < 0.001

Resveratrol, a phytochemical, has been considered as a good natural compound in the treatment of lymphomas but the underlying mechanism for anti-lymphomagenesis is largely unknown [47]. A recent study indicated that resveratrol could induce sphingolipid rheostat modulation (increased cellular ceramides/dihydroceramides but decreased S1P) [48]. To investigate the effect of resveratrol on S1P-YAP signaling, the protein levels of SPHK1 and YAP were analyzed in the tumor invaded LN tissues from EL4-WSHFD mice treated with resveratrol. Resveratrol treatment significantly attenuated the aberrant alterations of SPHK1 and phosphorylated YAP as well as YAP transcriptional targeting gene CTGF in the EL4-WSHFD mice (Fig. 7A). Resveratrol has also been reported as an immune booster to induce release of anticancer cytokines such as IFN-γ and stimulate polarization of macrophages against cancer [49]. Flow Cytometry analysis was therefore performed in the PMM from the peritoneal injection model of EL4-WSHFD mice to investigate the effect of resveratrol on macrophage polarization. Significantly increased subpopulation of F4/80^+^MHCII^+^ cells but decreased subpopulation of F4/80^+^CD206^+^ cells were found in EL4-WSHFD mice either treated with resveratrol or with IFN-γ, indicating that resveratrol had a profound effect on macrophage polarization, reprogramming the macrophages from an M2 to an M1 phenotype (Fig. 7B). These results encouraged us to further investigate whether resveratrol treatment could enhance immunotherapy against lymphomagenesis. In the xenograft model, the EL4-WSHFD mice were treated with resveratrol in combination with a murine anti-PD-L1 antibody for 21 days (Fig. 7C). The results showed that combination of resveratrol and anti-PD-L1 antibody caused significant tumor regression, in comparison with resveratrol only (*p* < 0.05), with anti-PD-L1 antibody only (*p* < 0.001), and with saline controls (*p* < 0.001) (Fig. 7D).

## Discussion

In this study, we report that up-regulated S1P/SPHK signaling plays a critical role in the obesity-associated lymphomagenesis. The major signaling components of the S1P-YAP axis and S1P-ALOX15 mediated macrophage polarization as well as the potential working hypothesis are shown as a schematic diagram in Graphical Abstract.

Although progress had been made in elucidating the role of S1P in lymphomas, no study was done for its role in obesity-associated lymphomagenesis. Previously, obesity was reported as a sufficient cause for increased expression of S1P in solid tumors [50]. We found increased S1P production and up-regulated SPHK1 expression in human lymphomas and in obesity-lymphoma mice. Engraftment of EL4 cells in obese mice suggested that up-regulated S1P/SPHK signaling was critical to mediate an aggressive behavior of obesity-lymphomas. In lymphoma cells, S1P treatment showed that YAP could not be phosphorylated but translocated to nucleus, as evident by the decreased phosphorylated-YAP and increased YAP target gene CTGF, while blockage of S1P receptors significantly increased phosphorylated-YAP and inhibited the S1P-induced cell proliferation and migration. Our results agreed with the studies from others, 1) the S1P/S1PR1 axis was suggested for lymphomagenesis in the DLBCL patients [51]; 2) the S1P/S1PR3 axis promoted the nuclear translocation of YAP, which contributed to the formation of the YAP-c-MYC complex [35]; and 3) S1P treatment promoted the proliferation of primary cultured OECs mediated by S1PR1 [52]. All the data indicated that the S1P/S1PR1/S1PR3-YAP signaling could be an underlying mechanism contributing to aggressive behavior of tumor growth in obesity-lymphoma.

The intra-tumoral immunosuppressive signaling has been proposed for obesity’s pro-tumor effects [53]. S1P, as a modulator of immune plasticity, has been reported to influence the phenotype of macrophages [54, 55], rendering an immunosuppressive tumor microenvironment [56]. It, however, is unknown how the S1P-mediated macrophage polarization contributing to immune suppression. An important finding in current study is that S1P-ALOX15 signaling can mediate macrophage polarization towards TAMs via cell cross-talk causing immune suppression in the obesity-lymphomagenesis. The S1P-ALOX15 signaling mediated TAMs was supported by the results: 1) the recruited myeloid cells contributed to tumor-infiltrated macrophages which showed M2 phenotype with high level of 12/15-LOX; 2) S1P induced up-regulation of TAMs markers were significantly attenuated by either S1PR inhibitors or ALOX15 inhibitor; and 3) significant tumor regression in the 12/15-LOX^−/−^ mice with obesity-lymphoma was found in association with less-infiltration of M2-macrophages/TAMs. As a key enzyme in the synthesis of the pro-inflammatory lipid mediators, ALOX15 had been widely studied in cancers [45], but its role on tumor progression is controversial. With the enzymatic activities of ALOX15 to generate the inflammation-suppressing lipid mediators, it is not surprising for its role being reported to inhibit inflammation-driven tumorigenesis [57]. However, studies also have shown that tumorigenesis is strengthened by activating the ALOX15 [23, 58]. Unfortunately, our understanding of the role of macrophage-ALOX15 in TAMs is even more obscure [59]. Because macrophage is a major cellular player in metabolic reprogramming and represent an important target of fatty acid metabolites in metabolic disorder [60, 61], the results from current study called attention to further investigation of the S1P-ALOX15 signals in macrophages, not only for understanding obesity-lymphomagenesis but also for finding the potential strategy of immune checkpoint blockade therapy. Based on its bioactivities for S1P reduction and immune modulation, resveratrol was used to treat obesity-lymphoma mice, and the results showed profound anti-lymphomagenetic effects, via down-regulating S1P-YAP axis and modulating polarization of macrophages. In addition, resveratrol significantly enhanced the therapeutic efficacy of anti-PD-L1 antibody in the obesity-lymphoma mice. Our data suggested that S1P-targeted therapy could be potentially effective and immune-enhancive against obesity-lymphomagenesis.

In conclusion, S1P/S1PR initiated the feedback loops, whereby S1P-S1PR1/S1PR3-YAP signaling mediated lymphomagenesis contributed to tumor aggressive growth, and S1P-ALOX15 signaling mediated TAMs contributed to immunosuppressive microenvironment in obesity-lymphoma. S1P-targeted therapy was potentially effective and immune-enhancive against obesity-lymphomagenesis.

## Methods

### Cells, lymphoma tissue samples, and human lymphoma cohort

A murine T lymphocyte (lymphoma) line EL4 (ATCC® TIB-39™), a human T lymphocyte (lymphoma) line HH (ATCC® CRL-2105™), a human B lymphocyte (lymphoma) line SU-DHL-4 (ATCC® CRL-2957™), and a human monocyte (Acute monocytic leukemia) line THP-1 (ATCC® TIB-202™) were purchased from American Type Culture Collection (ATCC) (Manassas, VA). HH, SU-DHL-4 and THP-1 cells were cultured in ATCC-formulated RPMI-1640 Medium (ATCC 30–2001) with 10% FBS (SigmaAldrich, MO) and Pen/Strep (Corning Cellgro). EL4 cells were cultured in ATCC-formulated Dulbecco’s Modified Eagle’s Medium (ATCC 30–2002) with 10% horse serum (SigmaAldrich, MO) and Pen/Strep (Corning Cellgro). The formalin-fixed paraffin-embedded (FFPE) tissue sections of human normal lymph node (Bio Chain, T2234161) were purchased from Biotrend (Miramar Beach, FL). The paraffin tissue sections of human lymphoma tissue array (XNHL060–01) were purchased from US Biolab (Rockville, MD). A cohort of 2094 lymphoma patients enrolled between 2011 and 2021 at the Cancer Center Bio-Repository of The First Hospital of Jilin University. The data for the lymphoma patients were extracted from the hospital health records by two onco-hematologists and validated by third onco-hematologist. Progression-free survival (PFS) and overall survival (OS) of three main subtypes of lymphomas (DLBCL, FL, PTCL) in this cohort were analyzed (Supplementary Method). The human procedures for this study were approved by the Institutional Review Board for Human Study at The First Hospital of Jilin University (#2021–690).

### Animals and obesity-lymphoma models

C57BL/6 J mice (Strain #000664) and 12/15-LOX KO mice (Strain #002778) were purchased from Jackson Laboratories (Bar Harbor, ME). The animals were housed four per cage, given rodent chow and tap water ad libitum, and maintained at 22 °C and on a 12-hour light/dark cycle. The wild type (WT) C57BL/6 J mice were crossed to 12/15-LOX KO mice to generate heterozygous F1 offspring. The F1 heterozygous mice were crossed to generate homozygous 12/15-LOX^−/−^ mice and 12/15-LOX^+/+^ WT littermates. The 12/15-LOX^−/−^ mice and WT littermates were utilized for establishment of obesity-lymphoma models. To induce obesity, the mice were fed with high-fat and high-fructose diet (WSHFD) (D16030909, 60% kcal fat, 19% kcal fructose, Research Diets, Inc., NJ) for 2 months, modified from our previous reports [62, 63], while 10% kcal fat diet (D12450B, Research Diets, Inc., New Brunswick, NJ) was used as control diet (CD). Food intake was recorded every day and body weight was measured every 2 weeks. According to the formula for calculating the degree of obesity of the mouse, degree of obesity (%) = (actual weight of the experimental group-average weight of the control group) / average weight of the control group × 100%. The degree of obesity greater than 20% was defined as obesity. Two obesity-lymphoma models were established by 1. xenograft and 2. peritoneal injection in the obese mice. For the xenograft model, EL4 cells (2 × 10^6^) were suspended in 0.1 ml PBS and injected subcutaneously into the right inguinal region of mice (6 mice per group). Tumor volume (mm^3^) was recorded every 2 days and calculated by the following formula: tumor volume = (length) × (width)^2^/2. Mice were sacrificed at the 21 days post-tumor xenograft, and blood, serum, tumor, lymph node and adipose tissues (gonadal, restroperitoneal and mesenteric) were collected for further analysis. For the peritoneal injection model, EL4 cells (1 × 10^6^) in 0.5 ml PBS were injected into peritoneal cavity. Mice were sacrificed at the 7 days post-peritoneal injection and peritoneal cavity was repeatedly washed using PBS to collect the peritoneal exudate cells according to a previous report for murine macrophage isolation [64]. The collected cells were suspended in Percoll® Cytiva Solution (17–0891-01, Sigma-Aldrich) to perform percoll centrifuge to separate the peritoneal monocytes/macrophages from the injected tumor cells for Flow Cytometry assay. All animal procedures were approved by the Institutional Animal Care and Use Committee of the University of Louisville, which is certified by the American Association for Accreditation of Laboratory Animal Care.

### Treatments

For in vitro studies, the concentrations (100 nM, 1 μM) of S1P were selected based on a colorimetric XTT assay (supplemental file, Fig. S4A) to study the effects of S1P on cell proliferation and cellular signal transduction in the lymphoma cells (HH cells and SU-DHL-4 cells). The S1PR inhibitors including W146, CAY10444, and FTY720 were used at 10 μM based on pilot studies. To determine the effects of S1P on macrophage polarization, phorbol 12-myristate 13-acetate (PMA) was used at 10μg/ml for 48 hours to stimulate M0 polarization of THP-1 cells. Lipopolysaccharide (LPS, from *Escherichia coli*, Sigma Aldrich, USA) was used of at 1 μg/mL for the purpose to induce M1 polarization. S1P was sued at 100 nM, 1 μM, 5 μM, and 10 μM to induce M2 polarization. For in vivo study of resveratrol and IFN-γ treatments in the peritoneal injection model, resveratrol (R5010, Sigma-Aldrich) (0.2 mg/kg, i.p.) was used administrated daily and mouse recombinant IFN-γ (IF005, Sigma-Aldrich) (40 μg/kg, i.p.) was used administrated daily, up to 7 days. For in vivo study of resveratrol treatment and anti-PD-L1 therapy in the xenograft model, resveratrol (0.2 mg/kg, i.p.) was administrated daily and a murine anti-PD-L1 antibody (BioXcell; Item No. BE0101) (10 mg/kg, i.p.) administrated every three at day, up to 21 days.

### Statistical analysis

For PFS and OS analysis in human lymphomas, Mann–Whitney U test was used to compare the medians between groups. Categorical variables were expressed as numbers and percentages, while the Fisher exact test was used to compare the percentages. For in vitro studies, experiments were performed with a minimum of triplicate samples and triplicate repetition of experiments. Statistical analysis and graphics were performed by using GraphPad Prism 7.00 software (San Diego, California). Statistical significance was determined by ANOVA. The post hoc Tukey’s test was used for analysis of any differences between groups. The collected data from experiments were presented as mean ± SD. Group difference was considered significant for *P* < 0.05 (*), *P* < 0.01(**), *P* < 0.001(***).

## Supplementary Information

**Additional file 1.**

## Data Availability

Not applicable.
