# Supplementary material for: S1PR1/S1PR3-YAP signaling and S1P-ALOX15 signaling contribute to an aggressive behavior in obesity-lymphoma
Source: J Exp Clin Cancer Res. 2023 Jan 5;42:3. doi: 10.1186/s13046-022-02589-7 (PMC9814427; doi:10.1186/s13046-022-02589-7)
Supplement: Supplementary file 1 — Additional file 1. [file 13046_2022_2589_MOESM1_ESM.docx]

**Supplementary materials**

Table 1. List of Antibodies

| **Antibody** | **Source (catalog)** |
| --- | --- |
| S1P | Abcam (ab224618) |
| SPHK1 | Abcam (ab71700) |
| SPHK/SPHK1(phospho-Ser225) | LSBio (LS-C26925) |
| S1P1/EDG1 | Abcam (ab11424) |
| EDG3/S1P3 | Abcam (ab108370) |
| Cyclin D1 | Cell Signaling Technology (cst2978) |
| c-Myc | Cell Signaling Technology (cst5605S) |
| E Cadherin | Abcam (ab76055) |
| Vimentin | Abcam (ab92547) |
| VEGF Receptor 1 | Abcam (ab32152) |
| Ki67/MKI67 | Novus Biologicals (NB500-170SS) |
| CD11b | Abcam (ab184308) |
| Ly6c | Abcam (ab15627) |
| CD206(Mannose Receptor) | Abcam (ab64693) |
| F4/80 | Abcam (ab6640) |
| Phospho-YAP (Ser127) | Cell Signaling Technology (cst13008S) |
| YAP | Cell Signaling Technology (cst14074S) |
| Anti-15 Lipoxygenase 1 | Abcam (ab244205) |
| Arginase-1 | Cell Signaling Technology (cst 93668S) |
| TGF-β | Cell Signaling Technology (cst3711S) |
| Donkey Anti-Rabbit IgG H&L (Alexa Fluor® 488) | Abcam (ab150061) |
| Goat Anti-Rat IgG H&L (Alexa Fluor® 594) | Abcam (ab150160) |
| GAPDH | Cell Signaling Technology (cst5174S) |
| β-Actin | Cell Signaling Technology (cst3700S) |
| Phospho-HSL (Ser563) | Invitrogen (PA5-17488) |
| Phospho-HSL (Ser660) | Cell Signaling Technology (cst4126S) |
| HSL | Abcam(ab45422) |
| IRS | Abcam(ab245314) |
| MPO | Abcam(ab208670) |
| FASN | Abcam(ab22759) |
| CD36 | Abcam(ab133625) |
|  |  |

| **Antibody** | **Source (catalog)** |
| --- | --- |
| Rat Anti-Mouse CD11b-FITC | SouthernBiotech (1561-02) |
| APC anti-mouse Ly-6C Antibody | Biolegend (128016) |
| PE Rat Anti-Mouse F4/80 | BD Pharmingen (565410) |
| APC anti-mouse CD206 | Biolegend (141708) |
| FITC Rat Anti-Mouse CD8a | Biolegend (553031) |
| APC Rat Anti-Mouse CD4 | BD Pharmingen (553051) |

Table 2. List of primers

| **Mouse** | | **Gene**  **ID** | | **Sequence (5‘-3’) mouse** | | |  | | **Product**  **Length** |
| --- | --- | --- | --- | --- | --- | --- | --- | --- | --- |
| Mrc1 | | 17533 | | Forward | | 5'- TTGCACTTTGAGGGAAGCGA -3' | | | 102 |
| (Cd206) | |  | | Reverse | | 5'- CCTTGCCTGATGCCAGGTTA -3' | | |  |
| Arg1 | | 11846 | | Forward | | 5'- CAGATATGCAGGGAGTCACC -3' | | | 250 |
|  | |  | | Reverse | | 5'- CAGAAGAATGGAAGAGTCAG -3' | | |  |
| Chi3l3 | | 12655 | | Forward | | 5'-TTTGTCCTTAGGAGGGCTTCCTC-3' | | | 437 |
|  | |  | | Reverse | | 5'-TCACAGGTCTGGCAATTCTTCTG-3' | | |  |
| Retnla | | 57262 | | Forward | | 5'-ACCTCTTCACTCGAGGGACAGTTGGCAGC-3' | | | 294 |
|  | |  | | Reverse | | 5'-GTCCCAGTGCATATGGATGAGACCATAGA-3' | | |  |
| IL-10 | | 16153 | | Forward | | 5'-GCACCTGCTTGACTCTGACATCTT-3' | | | 198 |
|  | |  | | Reverse | | 5'-CGAAGGATCTTGGAACGGAGAAGG-3' | | |  |
| TGFβ | | 21803 | | Forward | | 5'-CGTGGAAATCAACGCTCCAC-3' | | | 265 |
|  | |  | | Reverse | | 5'-CCACGTAGTAGACGATGGGC-3' | | |  |
| Nos2 | | 18126 | | Forward | | 5'- CCACCCGAGCTCCTGGAAC -3' | | | 70 |
|  | |  | | Reverse | | 5'- CCCTCCTGATCTTGTGTTGGA -3' | | |  |
| TNF-α | | 21926 | | Forward | | 5'- CGTGCTCCTCACCCACAC -3' | | | 133 |
|  | |  | | Reverse | | 5'- GGGTTCATACCAGGGTTTGA -3' | | |  |
| IL-1β | | 16176 | | Forward | | 5'- TCAGGCAGGCAGTATCACTCATT -3' | | | 75 |
|  | |  | | Reverse | | 5'- GGAAGGTCCACGGGAAAGA -3' | | |  |
| IL-6 | | 16193 | | Forward | | 5'- ACAACCACGGCCTTCCCTACTT -3' | | | 109 |
|  | |  | | Reverse | | 5'- GTGTAATTAAGCCTCCGACT -3' | | |  |
| IL12b | | 16160 | | Forward | | 5'- AACTTGAGGGAGAAGTAGGAATGG -3' | | | 180 |
|  | |  | | Reverse | | 5'- GGAAGCACGGCAGCAGAATA -3' | | |  |
| CXCL10 | | 15945 | | Forward | | 5'-CCAAGTGCTGCCGTCATTTT-3' | | | 177 |
|  | |  | | Reverse | | 5'-CTCAACACGTGGGCAGGATA-3' | | |  |
| 12/15-LO | | 11687 | | Forward | | 5'- CGTGGTTGAAGACTCTCAAGG -3' | | | 156 |
|  | |  | | Reverse | | 5'- CGAAATCGCTGGTCTACAGG -3' | | |  |
| Sptlc1 | | 268656 | | Forward | | 5'-TGTCCCCTTCCAGAACTGGTTA-3' | | | 121 |
|  | |  | | Reverse | | 5'-TCCCATAGTGCTCGGTGACT-3' | | |  |
| Cers1 | | 93898 | | Forward | | 5'-ACACACATCTTTCGGCCCCTG-3' | | | 173 |
|  | |  | | Reverse | | 5'-TAGAAGACAGAGGGCGGGTC-3' | | |  |
| Cers2 | | 76893 | | Forward | | 5'-GGCGCTAGAAGTGGGAAACG-3' | | | 75 |
|  | |  | | Reverse | | 5'-TCTGGAGCATCTCGGCGT-3' | | |  |
| Cers3 | | 545975 | | Forward | | 5'-GGGTGGGTCTCTGCAAGAAT-3' | | | 153 |
|  | |  | | Reverse | | 5'-GAAACATTCTTCTTCCCAAAGCG-3' | | |  |
| Cers4 | | 67260 | | Forward | | 5'-AAAGCAGGGATTGGGACCAC-3' | | | 105 |
|  | |  | | Reverse | | 5'-AACTCCTCGCTACAGAGCCT-3' | | |  |
| Cers5 | | 71949 | | Forward | | 5'-ATCAGGACAAGCCTCCAACG-3' | | | 112 |
|  | |  | | Reverse | | 5'-AACCAAGGCATCGACCAGAG-3' | | |  |
| Cers6 | | 241447 | | Forward | | 5'-GGTTTTGGCTTCCGCACAAT-3' | | | 152 |
|  | |  | | Reverse | | 5'-TGGTTTGGCTATGAATCTCTCG-3' | | |  |
| Degs1 | | 13244 | | Forward | | 5'-CCGCGGCTTATCGACTAGA-3' | | | 176 |
|  | |  | | Reverse | | 5'-GATACTTTGCTAAGATCTCCTTGCG-3' | | |  |
| Degs2 | | 70059 | | Forward | | 5'-TTACTACCTGCCACTGGTGCG-3' | | | 139 |
|  | |  | | Reverse | | 5'-ACACTTGCGCTTAACCCTGG-3' | | |  |
| Acer1 | | 171168 | | Forward | | 5'-TTCGGCATCTGATTGCGGTT-3' | | | 130 |
|  | |  | | Reverse | | 5'-AGGACGTGCCAA ATGCTGT-3' | | |  |
| Acer2 | | 230379 | | Forward | | 5'-GTTCTACAACACGATCAGCAATGT-3' | | | 158 |
|  | |  | | Reverse | | 5'-GGGTTGCATGGAAGTAGACG-3' | | |  |
| Acer3 | | 66190 | | Forward | | 5'-ACTGGTGCGAGGAGAACTAC-3' | | | 170 |
|  | |  | | Reverse | | 5'-CATTCCTACCACTGTGAGTGC-3' | | |  |
| Slc27a1 | | 26457 | | Forward | | 5'-ATGTGCTCTATGACTGCC-3' | | | 164 |
|  | |  | | Reverse | | 5'-TATGTACTGCACTACCGTG-3' | | |  |
| Slc27a2 | | 26458 | | Forward | | 5'-GAGGATACAAGATACCATTGAG-3' | | | 194 |
|  | |  | | Reverse | | 5'-GGAATATTCAGAGGTTCAGAG-3' | | |  |
| Slc27a3 | | 26568 | | Forward | | 5'-AATCTGAAACCTTCCACTTG-3' | | | 86 |
|  | |  | | Reverse | | 5'-ACAAAAGATACCCGAAAACC-3' | | |  |
| Slc27a4 | | 26569 | | Forward | | 5'-CTCAGCTATCTGTGAGATCC-3' | | | 172 |
|  | |  | | Reverse | | 5'-GAGCTTATCTGTAAAACCCTTG-3' | | |  |
| Slc27a5 | | 26459 | | Forward | | 5'-ACCTCTGTACCATACGATAG-3' | | | 146 |
|  | |  | | Reverse | | 5'-CCACATACAAGATCACTGTTAC-3' | | |  |
| CD36 | | 12491 | | Forward | | 5'-CATTTGCAGGTCTATCTACG-3' | | | 182 |
|  | |  | | Reverse | | 5'-CAATGTCTAGCACACCATAAG-3' | | |  |
| Acads | | 11409 | | Forward | | 5'-CGTAGAGCTCTCGGTGTTCG-3' | | | 123 |
|  | |  | | Reverse | | 5'-GACCAACTCCTTCTCGGCAA-3' | | |  |
| Acadm | | 11364 | | Forward | | 5'-AAAAGAGCCTGGGAACTCGG-3' | | | 169 |
|  | |  | | Reverse | | 5'-GAATCACAGGCATTTGCCCC-3' | | |  |
| Acox1 | | 11430 | | Forward | | 5'-CATGTGGTTTAAAAACTCTGTGC-3' | | | 123 |
|  | |  | | Reverse | | 5'-GGCATGAAGAAACGCTCCTG-3' | | |  |
| Cpt1a | | 12894 | | Forward | | 5'-TGCCTCTATGTGGTGTCCAA-3' | | | 300 |
|  | |  | | Reverse | | 5'-CATGGCTTGTCTCAAGTGCT-3' | | |  |
| PPARα | | 19013 | | Forward | | 5'-GATGTCACACAATGCAATTC-3' | | | 107 |
|  | |  | | Reverse | | 5'-CAGTTTCCGAATCTTTCAGG-3' | | |  |
| Mttp | | 17777 | | Forward | | 5'-TGCAAAATAGCGGTCACACA-3' | | | 125 |
|  | |  | | Reverse | | 5'-TTTGTAGCCCACGCTGTCTT-3' | | |  |
| Apoa1 | | 11806 | | Forward | | 5'-GCACGTATGGCAGCAAGATG-3' | | | 149 |
|  | |  | | Reverse | | 5'-GATTCAGGTTCAGCTGTTGGC-3 | | |  |
| Dgat1 | | 13350 | | Forward | | 5'-TAGAAGAGGACGAGGTGCGA-3' | | | 237 |
|  | |  | | Reverse | | 5'-TCAGGATCAGCATCACCACAC-3' | | |  |
| Acat1 | | 110446 | | Forward | | 5'-CTGGGCGCAGGTTTACCTAT-3' | | | 182 |
|  | |  | | Reverse | | 5'-GGTGTTGCTCCTCTGCTCAT-3' | | |  |
| FASN | | 14104 | | Forward | | 5'-TTGGCCTACACCCAGAGCTA-3' | | | 245 |
|  | |  | | Reverse | | 5'-TTGTGGTAGAAGGACACGGC-3' | | |  |
| ACC1 | | 107476 | | Forward | | 5'-ATG CGA TCT ATC CGT CGG TG-3' | | | 224 |
|  | |  | | Reverse | | 5'-CCA GCC CAC ACT GCT TGT A-3' | | | 147 |
| ACC2 | | 100705 | | Forward | | 5'-TTC CCC AGC CAG CAG ATA GC-3' | | |  |
|  | |  | | Reverse | | 5'-CTT CAT GTA GCC ACG GGT CC-3' | | |  |
| PPARγ | | 19016 | | Forward | | 5'-AAAGACAACGGACAAATCAC-3' | | | 195 |
|  | |  | | Reverse | | 5'-GGGATATTTTTGGCATACTCTG-3' | | |  |
| SREBP1 | | 20787 | | Forward | | 5'-CAGCAGGTCCCAGTTGTACT-3' | | | 174 |
|  | |  | | Reverse | | 5'-GATGGTCCCTCCACTCACCA-3' | | |  |
| GAPDH | | 14433 | | Forward | | 5'-GTG AAG GTC GGT GTG AAC GGA TT-3' | | | 151 |
|  | |  | | Reverse | | 5'-CGT GAG TGG AGT CAT ACT GGA ACA T--3' | | |  |
|  | |  |  | |  | | |  | |
| **Human** | **Gene**  **ID** | | | **Sequence (5‘-3’) mouse** | | |  | **Product**  **Length** | |
| CD206 | 4360 | | | Forward | | 5'-AATGGCATGAAGCGGAGACA-3' | | 444 | |
|  |  | | | Reverse | | 5'-ATTCCAGAGAAGCTTGGCCC-3' | |  | |
| Arg1 | 383 | | | Forward | | 5'-ACGGAAGAATCAGCCTGGTG-3' | | 281 | |
|  |  | | | Reverse | | 5'-GTCCACGTCTCTCAAGCCAA -3' | |  | |
| IL-10 | 3586 | | | Forward | | 5'-AGACAGACTTGCAAAAGAAGGC-3' | | 148 | |
|  |  | | | Reverse | | 5'-TCGAAGCATGTTAGGCAGGTT-3' | |  | |
| TGFβ | 7040 | | | Forward | | 5'-ATG TCA CGG TTA GGG GCT C--3' | | 146 | |
|  |  | | | Reverse | | 5'-GGC TTG CAT ACT GTG CTG TAT AG-3' | |  | |
| GAPDH | 2597 | | | Forward | | 5'-GTGAAGGTCGGTGTGAACGGATT-3' | | 151 | |
|  |  | | | Reverse | | 5'-CGTGAGTGGAGTCATACTGGAACAT-3' | |  | |

Table 3: Information of Tissue Array

| **Gender** |  | **Subtypes** |  |
| --- | --- | --- | --- |
| Male | 40 | DLBCL | 27 |
| Female | 20 | CLL/SLL | 3 |
|  |  | FL | 2 |
| **Age** |  | MALT | 12 |
| ＞60 | 14 | LPL | 2 |
| ≤ 60 | 46 | PTCL | 2 |
|  |  | AITL | 1 |
| **Involvement** |  | ALCL | 5 |
| Nodal involvement | 37 | EATL | 1 |
| Extranodal involvement | 24 | Other B-cell lymphomas | 3 |
| Bone | 1 | Other T-cell lymphomas | 2 |
| CNS | 3 |  |  |
| Intestine | 15 |  |  |
| Testis | 3 |  |  |
| Parotid gland | 1 |  |  |
|  |  |  |  |

Distribution of the subtypes of lymphoma according to the World Health Organization classification: DLBCL: Diffuse large B-cell lymphoma; FL: Follicular lymphoma; CLL/SLL: Chronic lymphocytic leukemia/Small lymphocytic lymphoma; MALT: Mucosa-associated lymphoma; LPL: Lymphoplasmacytic lymphoma; PTCL: Peripheral T-cell lymphoma; AITL: angioimmunoblast T-cell lymphoma; Adult T-cell lymphoma; ALCL: Anaplastic large cell lymphoma; EATL: Enteropathy-Associated T-Cell Lymphoma; CNS: Central Nervous System.

**Supplementary Methods**

Retrospective analysis in a cohort of 2094 lymphoma patients

**Study participants, setting and design:** We reviewed the cases for 2094 patients who were diagnosed with lymphomas in the First Hospital of Jilin University from 2011 to 2021. The data were extracted from the Hospital Health Records by two onco-hematologists and were validated by another onco-hematologist. Patients were categorized into high Body Mass Index (BMI, defined as BMI ≥ 25) group and low BMI (defined as BMI<25) group. Survival data were follow-up till the end of May 2021 and the disease progression was assessed. Three main subtypes of lymphomas (namely DLBCL, FL, PTCL) in this cohort were selected to analyze progression-free survival (PFS) and overall survival (OS).

**Statistical analysis:** We expressed continuous variables as medians (P25, P75). We employed the Mann–Whitney U test to compare the medians between groups. Categorical variables were expressed as numbers and percentages. We used the Fisher exact test to compare the percentages. In the time-to-event analysis, we used PFS and OS as the outcomes; we excluded patients who died during the follow-up. We used GraphPad Prism 7.00 software (San Diego, California) and R version 3.6.3 (University of Auckland, Oakland, New Zealand) for data analysis. All tests were 2-sided and a value of p < 0.05 was considered as statistically significance.

Gene Expression Omnibus (GEO) and Tumor Immune Estimation Resource (TIMER) analysis

Using GEO database (<https://www.ncbi.nlm.nih.gov/geo/>), four independent patient cohort datasets, 1) GSE56315 (DLBCL); GSE65135 (FL); 3) GSE132550 (PTCL-NOS); and 4) GSE59307 (CTCL) were obtained. We analyzed the gene expressions (SPHK1, SPHK2, and ALOX15) and prognostic relevance in the lymphoma patients. Differentially expressed genes (DEGs) between lymphomas and the respective controls were identified by R software version 3.4.1 (<https://www.r-project.org/>) and LIMMA (Liner models for microarray data) package. This work applied adjust p < 0.05 and |log Fold Change (FC)|> 1 as upregulation and p < 0.05 and |log FC|< -1 as downregulation. We also explored the correlation between survivals and six immune cells (B cells, CD4+ T cells, CD8+ T cells, neutrophils, macrophages, and dendritic cells) in the TMER of DLBCL. The TIMER database (https://cistrome.shinyapps.io/timer/), which collected 24 samples with 3 dying in DLBCL from TCGA. We set the Split Percentage of Patients is 25% and get the KM curve. The difference at a P-value < 0.05 was considered to be statistically significant in macrophage.

Metabolic cage

The mice were monitored for VO_2_ (mL/kg/h) and VCO_2_ (mL/kg/h) using metabolic cages (TSE Phenomaster/Labmaster Caging System, TSE Systems, Inc., Chesterfield, MO). In brief, mice were acclimated to the apparatus for 16 hours before data collection, commenced and allowed access to their respective diet and water ad libitum. The data were collected approximately for 24hours. Energy expenditure and RER (VCO_2_/VO_2_) was calculated as daytime (light, 12hours) and nighttime (dark, 12 hours).

Triglyceride assay

The level of triglycerides from plasma and tissue homogenates was determined using a colorimetric assay kit from Cayman (#10010303). Briefly, 10 µl of sample or standards (serial dilutions of 8 standards from 0 to 200 mg/dl) was mixed with 150 µl of Enzyme Mixture Solution, and incubated for 60 minutes at room temperature. The absorbance at 540nm was measured using a plate reader.

ELISA assay for S1P

The level of S1P from plasma and tissue homogenates was determined using an ELISA assay kit from Abclonal (#RK00714). Briefly, 50 µl of sample or standards (serial dilution of 6 standards from 0 to 1000 ng/ml) was mixed with 50 µl of Standard/sample Diluent and 50 µl of Biotin conjugate Antigen Working Solution in the 96-well plate. The mixture was incubated for 1 hour at 37 °C. After washing 3 times with 350 µl of Wash Buffer, 100 µl of Streptavidin-HRP Working Solution was added and incubated for 30 min at 37 °C. After 5 washes, 90 µl of TMB Substrate was added and incubated for 20 min at 37 °C. After 50 µl of Stop Solution was added, the optical density at 450mn was determined within 5 min. Wavelength correction was made at 620nm.

Flow Cytometry

Flow Cytometry assay was performed in single-cell suspension of isolated mouse peripheral blood mononuclear cell (PBMC) and peritoneal monocyte/macrophage (PMM). In brief, the cells were blocked with TruStain FcX anti-mouse CD16/32 antibody (#101319, BioLegend, San Diego, CA) for 5 min, then stained with Rat Anti-Mouse CD11b-FITC and APC anti-mouse Ly-6C, PE Rat Anti-Mouse F4/80 and APC anti-mouse CD206, FITC Rat Anti-Mouse CD8a and APC Rat Anti-Mouse CD4 antibodies for 20 min at 4 °C. eBioscience Fixable Viability Dye (#65-0865-14, ThermoFisher, Waltham, MA) was added together with the above antibodies to stain for dead cells. Cells were washed with cell staining buffer (#420201, BioLegend, San Diego, CA), followed by incubation with fixation buffer (#00-8222-49, eBiosciences, USA) and permeabilization buffer (#00-8333-56, eBiosciences, USA) according to the manufacturer’s instructions. After washing with permeabilization buffer, the cells were resuspended in permeabilization buffer for flow cytometry. Flow cytometry data were then collected using a FACSCalibur (BD Pharmingen) and analyzed using FlowJo X software (vX0.7, Tree Star, San Carlos, CA) to determine macrophage polarization. The pre-gating strategies are based on CD45+CD11b+ for macrophages, CD45^+^CD11b^+^Ly-6C^-^F4/80^+^MHC-II^+^ for macrophage M1 polarization, CD45^+^CD11b^+^Ly-6C^-^F4/80^+^CD206^+^ for macrophage M2 polarization.

Immunofluorescence staining of mouse tumor tissue monocytes/macrophages:

Frozen tissue section was used for immunofluorescence staining. In brief, the tissue sections were washed with pre-cooled PBS and add 1% paraformaldehyde fixative solution to fix at room temperature for 10 minutes. After washing with PBS, 1% BSA+PBS containing 5% goat serum and 5% donkey serum were used for blocking for 30 minutes at room temperature. The antibodies, CD11b (ab184308 1:500), Ly6C (ab15627 1:100), F4/80 (ab6640 5ug/ml), and CD206 (ab64693 1ug/ml) were applied and incubated overnight at 4°C. After re-washing, the tissue sections were incubated with corresponding species of secondary antibody (1:1000) at room temperature and avoid light for 1 hour. After counter staining with DAPI, digital images were acquired with the Olympus 1x51 microscope (Olympus, Pittsburgh, PA) using the Olympus DP72 digital camera and measured via the cellSense Dimension imaging system (Olympus Life Science, Tokyo, Japan). Computer image analysis of staining was performed, and the acquired color images were defined and quantified per software specifications with the antibody expression.

RNA extraction and real-time–polymerase chain reaction (RT-PCR)

Total RNA was extracted using the TRIzol reagent (Invitrogen, CA). First-strand complimentary DNA (cDNA) was synthesized from total RNA according to the kit protocol provided by manufacturer (Promega, Madison, WI). Quantitative PCR was carried out using a kit of Powerup SyberGreen Master Mix (Applied Biosystems, Carlsbad, CA) and the ABI 7300 real-time PCR system (Applied Biosystems, Carlsbad, CA). A list of primers is shown in the Table 2 of supplemental file. The 2^-ΔΔCt^ method was used to determine gene quantification with GAPDH or β-actin used as an endogenous references.

Western blot analysis

The protein levels were semi-quantified by Western blot analysis. In brief, electrophoresis was performed on 12% SDS-PAGE gel and the proteins were transformed to nitrocellulose membrane. The membranes were incubated with the primary antibodies (see Table 1 antibody list in supplemental file) overnight at 4$℃$ and with secondary antibody for 1 huor at room temperature. The antigen-antibody complex was then visualized using ECL kit (Amersham, Piscataway, NJ). The protein bands were quantified by densitometry analysis and protein expression was presented pixel ratio of target protein vs endogenous reference, GAPDH or β-actin.

Immunohistochemistry staining analysis

Immunohistochemical staining was carried out on the paraffin-embedded material using the DAKO EnVision+System Kit. In brief, the sections were deparaffinized and hydrated. The slides were washed with a TRIS-buffer, and peroxidase blocking was performed for 5 minutes. After rewashing, the primary antibodies were applied for 60 minutes and then incubated with labeled polymer for 30 min at room temperature. The substrate-chromogen solution (diaminobenzidine) was added as a visualization reagent. Digital images were acquired with the Olympus 1×51 microscope (Olympus, Pittsburgh, PA) at 20x magnification using the Olympus DP72 digital camera via the cellSens Dimention imaging system. The procedure for the computer image analysis was performed, and the acquired color images from the immunohistochemical staining were defined a standard threshold according to the software specification. The computer program then quantified the threshold area represented by color images. Protein expressions were defined by the percentages of threshold area in acquired color.

XTT cell viability assay

In brief, the cells were seeded at 5×10^4^/ml and cultured with the respective medium in 96-well plate in an incubator (37°C, 5% CO_2_). The S1P concentrations were selected as 0 nM, 50 nM, 100 nM, 1 µM, 5 µM, 10 µM, and 20 µM in 100 µl medium to treat the cells for 12 hours, 24 hours, 48 hours, and 72 hours. After treatments, 20 µl XTT mixture was added into each well, mixed, and incubated at 37°C for 4 hours. The optical density value was read using a microplate reader at the wavelength of 450 nm.

Cell migration assay

In brief, the lymphoma cells were treated with serum-free medium for 12 hours, then treated with S1P and S1PR inhibitors for 6 hours. A 8-μm membrane insert (BD Biosciences, 354,480) which precoated with rat tail collagen was used to perform the migration assay. The treated cells were transferred into the insert at 2×10^6^ in 200ul serum-free medium. The insert was then placed in 24-well plate containing the culture media containing 10% FBS as chemoattractant. After 24 hours, the inserts were washed with PBS and fixed in methanol. After staining with crystal violet (0.05% w/v in methanol), the bottom surfaces of the stained inserts were then observed under a light microscope, and the numbers of stained cells were counted in 5 fields/insert. The cell migration ability was calculated based on the numbers of stained cells.

Co-culture assay

In brief, THP-1 cells were treated with 10 µg/ml phorbol 12-myristate 13-acetate (PMA) for 48 hours to stimulate M0 polarization and then seeded at 1 × 10^5^ in the insert of Trans-well plate for 24 hours. The insert was put on top of 24-well plate to co-cultured with lymphoma cells with or without S1P treatment for additional 24 hours.

**Supplementary Figures**


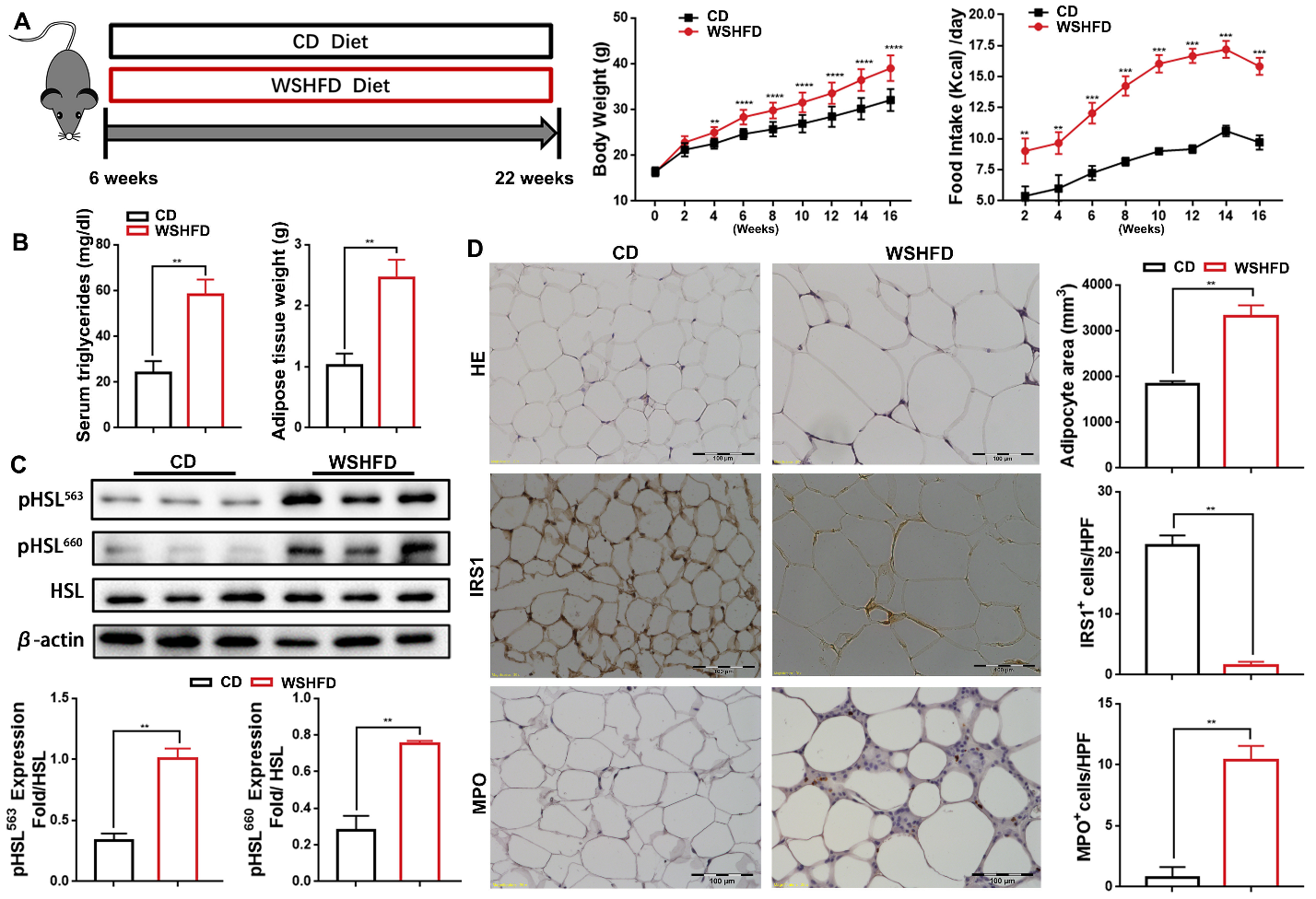


**Figure S1.** **A:** The changes of body weight and food intake calories in the mice during 16 weeks feeding of CD and WSHFD**. B:** The serum triglycerides and adipose tissue weight in CD mice and WSHFD mice at sacrifice. **C:** Western blot analysis for phosphorylated HSLs (ser563, ser660) and HSL in CD mice and WSHFD mice**. D:** The adipocyte area was measured by H&E staining, immunohistochemical (IHC) staining of IRS1 for insulin resistance, and MPO for inflammatory infiltration were determined in adipose tissue sections from CD mice and WSHFD mice. HSL: hormone-sensitive lipase; IRS1: insulin receptor substrate 1; MPO: Myeloperoxidase. *, *P* < 0.05; **, *P* < 0.01. ***, P < 0.001.


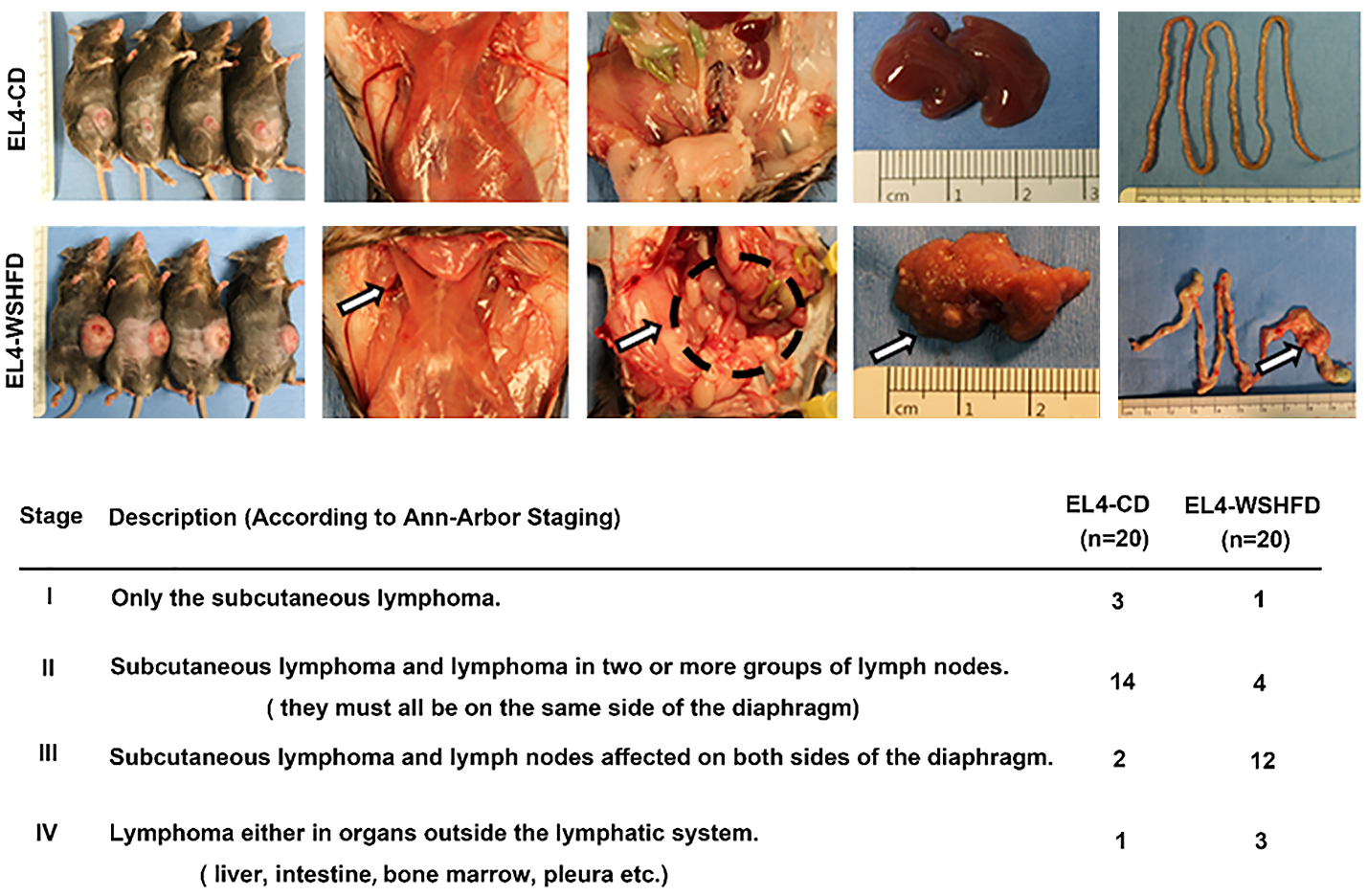


**Figure S2:** **Upper:** Representative gross anatomy of xenograft lymphoma in EL4-CD mice and EL4-WSHFD mice. Arrow: affected lymph nodes; lymphoma affected in liver and intestine. **Lower:** Simulate the clinical Ann-Arbor staging of lymphoma to compare the proportion of each stage of the two groups of mice (EL4-CD and EL4-WSHFD).


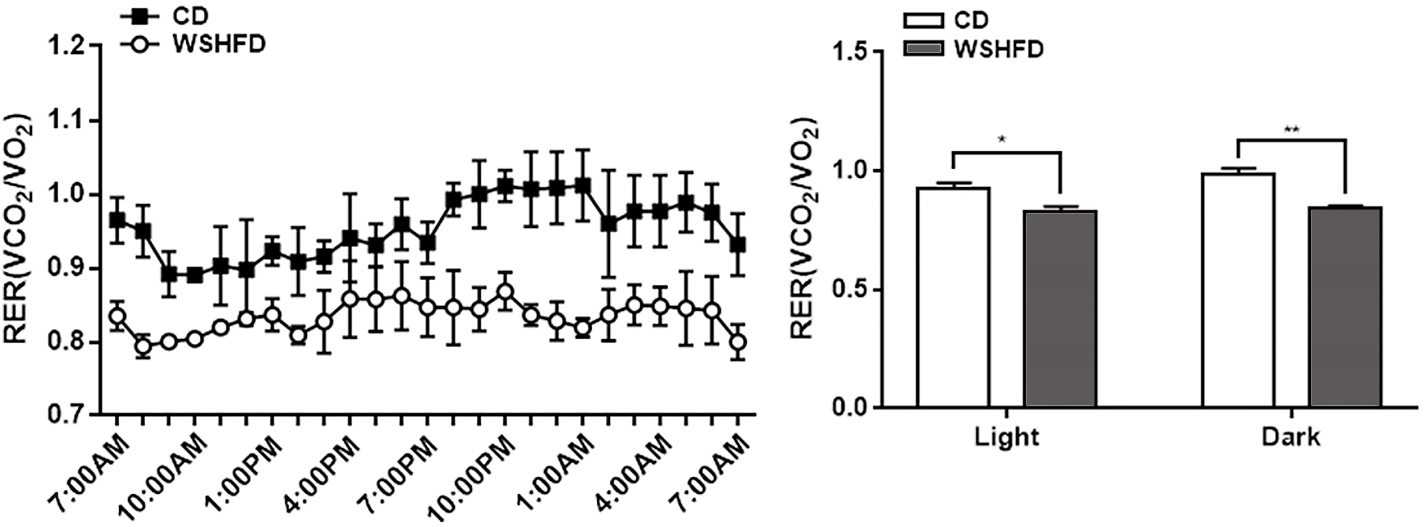


**Figure S3.** Use a metabolic cage to monitor VO_2_ (mL/kg/hour) and VCO_2_ (mL/kg/hour). The whole-body calorimetry was calculated as respiratory exchange ratio (RER). Whole-body calorimetry showed significantly decreased RER level in the WSHFD-feeding caused obese mice, compared to that in control mice, suggesting that less use of glucose and less gluconeogenesis contributed to aberrant lipid metabolism in the WSHFD-feeding caused obese mice*, *P* < 0.05; **, *P* < 0.01.


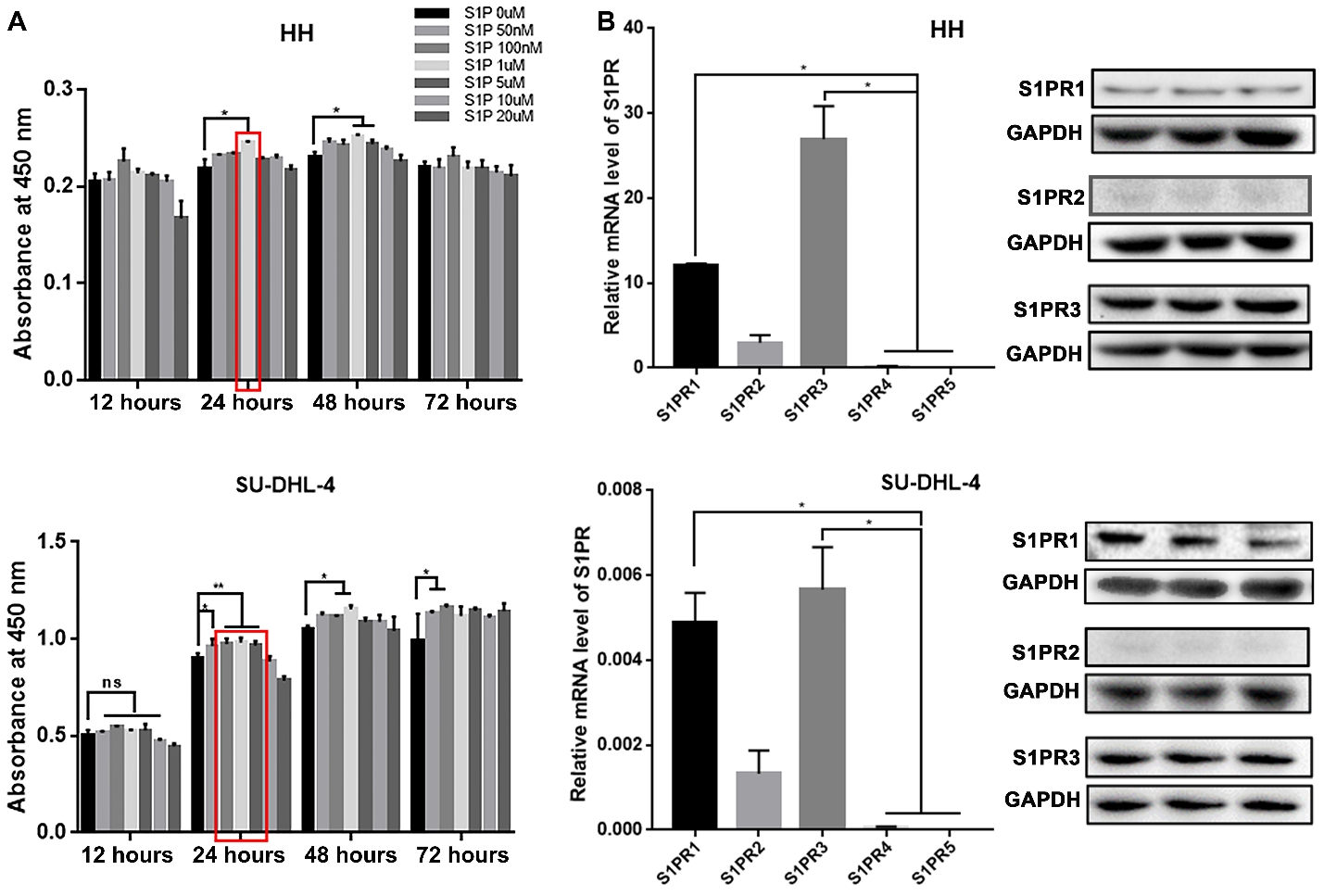


**Figure S4: A:** XTT assay was performed to determine the concentration and timepoint for S1P treatment. HH and SU-DHL-4 cells were treated with S1P 0 nM, 50 nM, 100 nM, 1 µM, 5 µM, 10 µM, 20 µM, for 12 hours, 24 hours, 48 hours, and 72 hours. Concentrations of 100 nM and 1 µM, and 24 hours were selected. **B:** A qPCR was performed to determine the mRNA levels of S1PR1-5 in HH cells and SU-DHL-4 cells. Western blot analysis for the protein levels of S1PR1-3 in the HH cells andSU-DHL-4 cells treated withS1P 0nM, 100 nM,1 µM for 24 hours. n s.: no statistical significance; *, *P* < 0.05; **, *P* < 0.01.

**
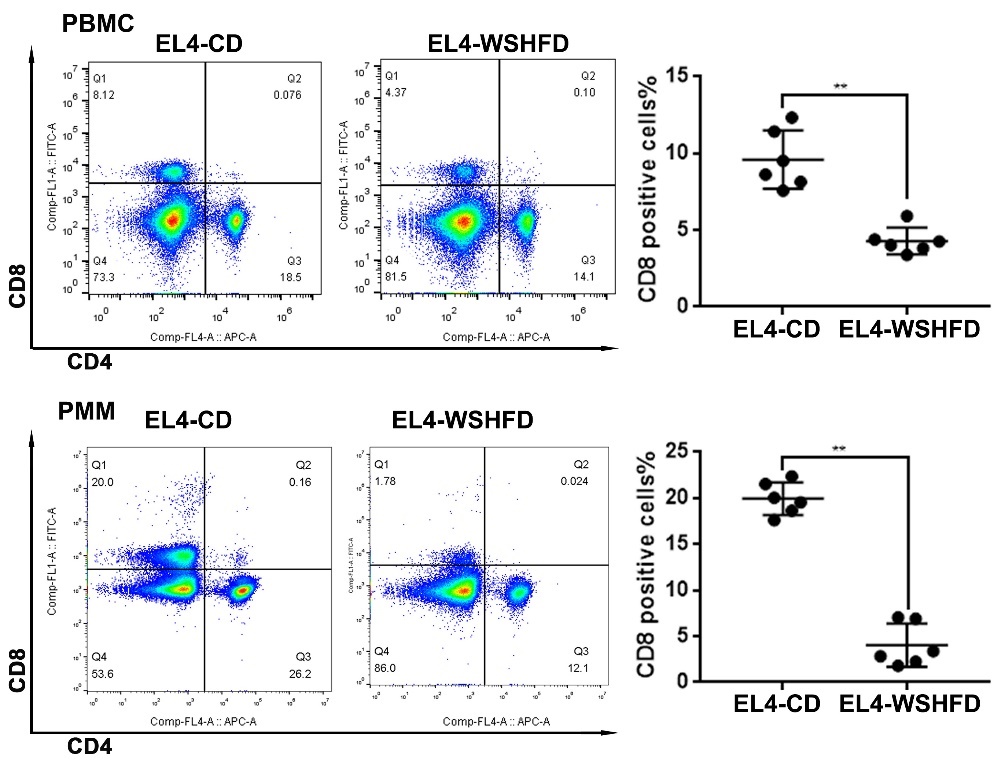
**

**Figure S5** Flow Cytometry analysis to detect CD8^+^ cells for potential M-MDSC derived TAMs in the collected PBMC and PMM from peritoneal injection model of EL4-WSHFD mice and EL4-CD mice.


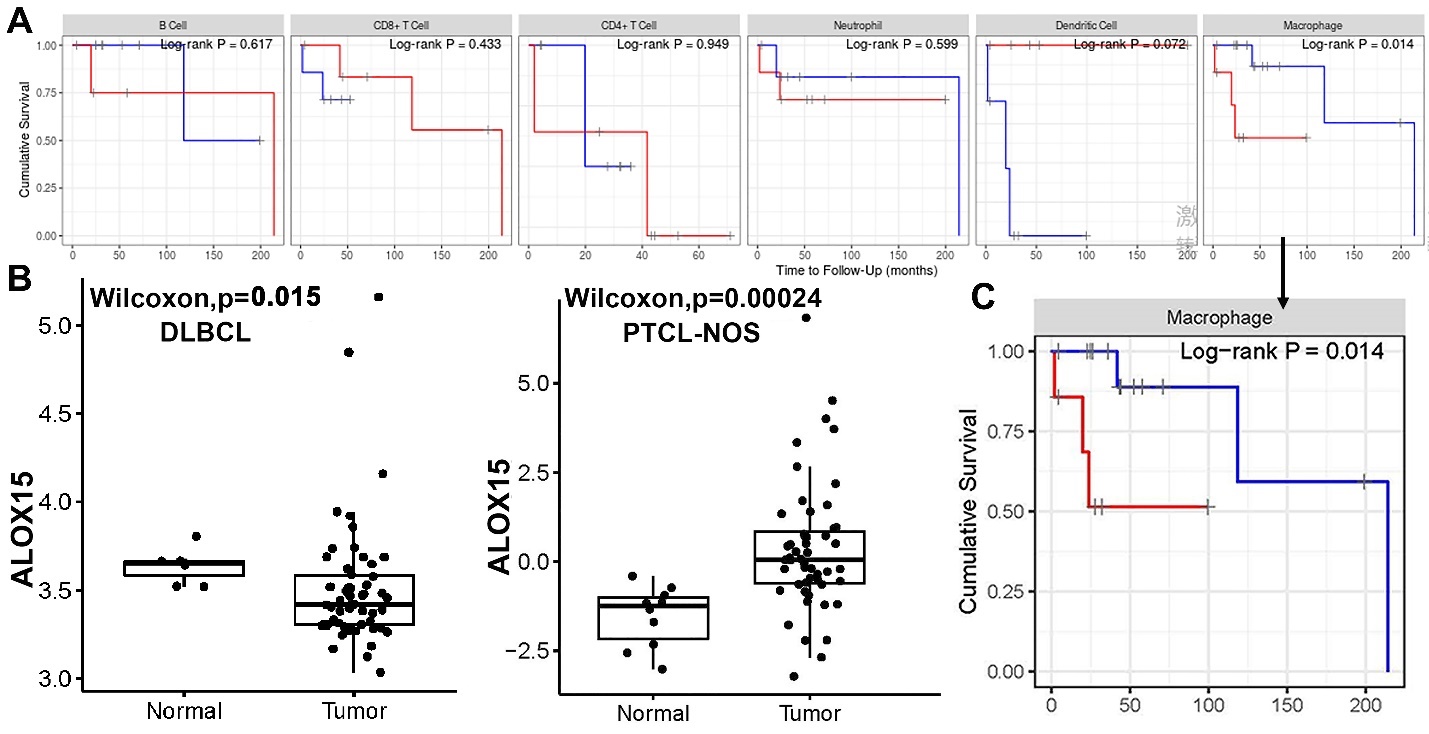


**Figure S6: A:** Using the Tumor Immune Estimation Resource (TIMER) database, the immune cell (B cells, CD4+ T cells, CD8+ T cells, neutrophils, macrophages, and dendritic cells) associated the survival rate was analyzed in the DLBCL by splitting 25% of the patients with high level of immune cells and 25% with low level of immune cells. **B:** Gene expression of ALOX15 was analyzed in DLBCL and PTCL-NOS patients using the GEO database. **C:** the KM curve showed a poor outcome for the patient with high level of macrophages.


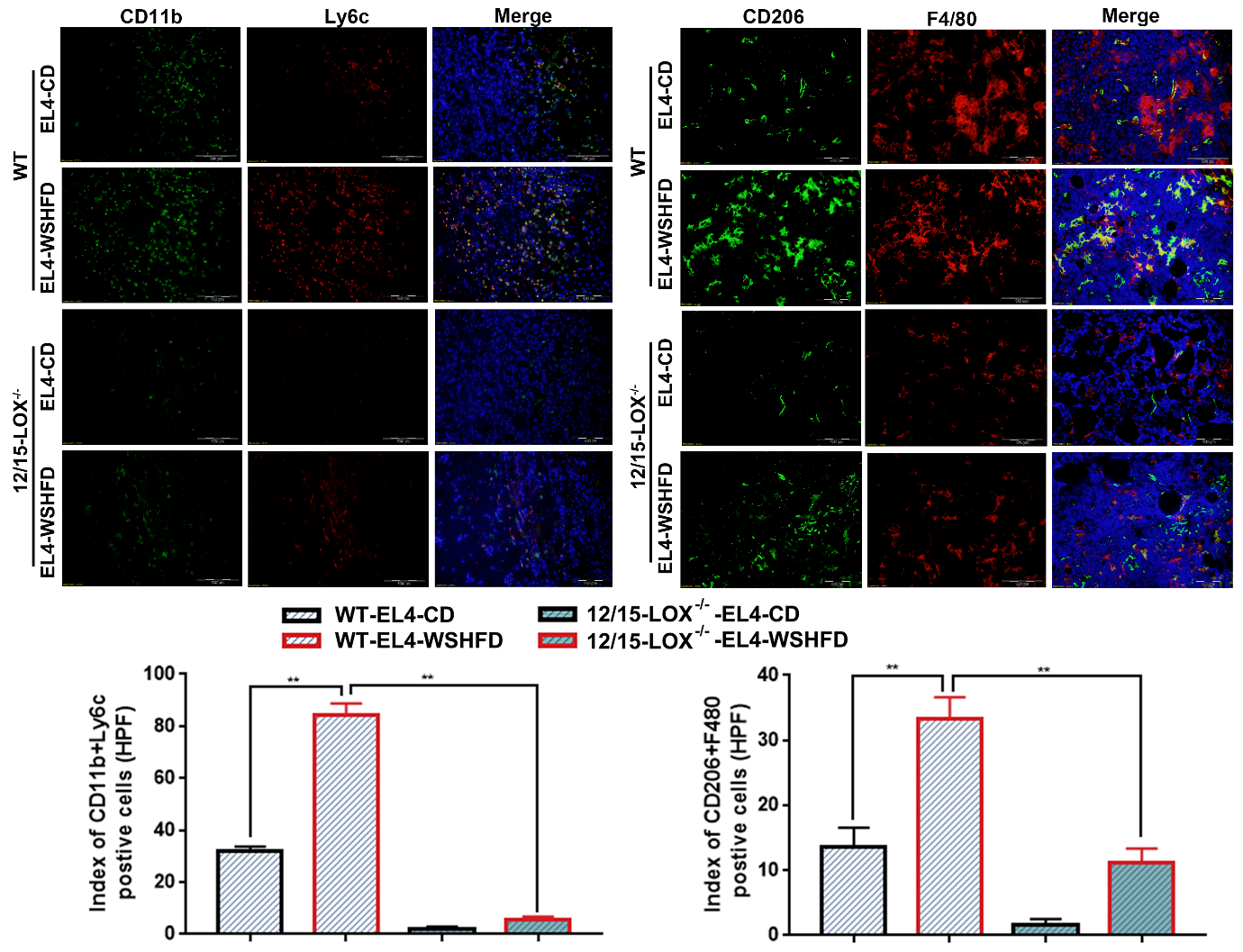


**Figure S7.** Dual immunofluorescent staining using the antibodies of anti-CD11b and anti-Ly6C as well as the antibodies of anti-CD206 and anti-F4/80 to detect the M-MDSC derived macrophages in the tissues of tumor invaded lymph node from xenograft model of WT-EL4-CD; WT-EL4-WSHFD Alox15^-/-^-EL4-CD; Alox15^-/-^-EL4-WSHFD. **, P < 0.01
